# Clinical Implications of Proton Pump Inhibitors and Vonoprazan Micro/Nano Drug Delivery Systems for Gastric Acid-Related Disorders and Imaging

**DOI:** 10.7150/ntno.100727

**Published:** 2024-09-30

**Authors:** Aseem Setia, Ranadheer Reddy Challa, Bhaskar Vallamkonda, Vaishali, Matte Kasi Viswanadh, Madaswamy S. Muthu

**Affiliations:** 1 Department of Pharmaceutical Engineering and Technology, Indian Institute of Technology (BHU), Varanasi-221005, India.; 2 Department of Pharmaceutical Science, School of Applied Sciences and Humanities, VIGNAN's Foundation for Science, Technology & Research, Vadlamudi-522213, Andhra Pradesh, India.; 3 Department of Pharmaceutics, KL College of Pharmacy, Koneru Lakshmaiah Education Foundation, Greenfields, Vaddeswaram 522302, AP, India.

**Keywords:** Gastric acid related disease, proton pump inhibitors, vonoprazan, imaging, case studies, clinical trials

## Abstract

Excessive stomach acid or bacterial infection are the root causes of gastric acid-related disorders, such as peptic ulcer disease and gastroesophageal reflux disease. Proton pump inhibitors including lansoprazole, omeprazole, esomeprazole, rabeprazole, etc. are medications used to treat gastric acid-related diseases. One of the most effective drugs for treating gastroesophageal reflux disease is vonoprazan, owing to its ability to strongly inhibit gastric acid. Proton pump inhibitors and vonoprazan work in distinct ways to prevent the production of stomach acid. Vonoprazan inhibits acid secretion by blocking the potassium-competitive acid blocker receptor, whereas proton pump inhibitors function by irreversibly blocking the proton pump in the parietal cells of the stomach. Delayed release tablets, delayed release capsules, minitablets, pellets, bilayer, floating, mucoadhesive tablets and nanoparticles, are some of the methods used in the development of micro/nano formulations with proton pump inhibitors and vonoprazan. Diagnosis and therapy of gastric acid-related illnesses, particularly those treated with drugs such as vonoprazan and proton pump inhibitors, rely heavily on imaging modalities such as CT scans, X-rays, endoscopy, fluorescence and HRM imaging. This review provides a comprehensive update on various micro/nanoformulations of proton pump inhibitors and vonoprazan. Moreover, we provide an outlook on clinical imaging of proton pump inhibitors and vonoprazan formulation for gastric acid related diseases. We have limited our discussion to case studies and clinical trials on proton pump inhibitors and vonoprazan for gastric acid related disease.

## Introduction

Gastric acid secretion inhibitors are effective medications that treat gastroesophageal reflux disease (GERD), peptic ulcers, and infections caused by helicobacter pylori 1. The severity of the situation surrounding diseases related to gastric acid has significantly improved ever since proton pump inhibitors (PPIs) were introduced to the market in the late 1980s. PPIs have traditionally been the primary treatment of choice for gastroesophageal reflux disease. Indeed, PPIs are effective in patients with both oesophageal reflux disease and non-oesophageal reflux disease (NERD) 2. Although PPIs are frequently utilized in clinical settings, the typical dose of PPIs does not always result in adequate suppression of stomach acid in all individuals due to the pharmacological limits of these medications 3. It has been observed that attaining total symptomatic relief with PPIs is more difficult than just mending mucosal breaks. This is the reason why approximately one-third of patients who have GERD are considered to be dissatisfied with the treatment that is currently being administered.

A series of medications known as PPIs are characterized by their primary effect of blocking the H+/K+-ATPase enzyme system that is located on the surface of parietal cells. This results in a significant and long-lasting reduction in the generation of stomach acid 4. The prodrugs known as PPIs are activated by acid and utilize a disulfide link to form a covalent interaction with the H+/K+-ATPase in the stomach. The first PPIs was introduced is omeprazole, which was then followed by lansoprazole, rabeprazole, pantoprazole, and esomeprazole 5. Compared to histamine H2 receptor antagonists (H2RAs), PPIs have a more potent inhibitory impact on the secretion of stomach acid 6, 7. Extensive metabolizers, intermediate metabolizers, ultra-rapid metabolizers, and poor metabolizers were the four categories that were used to categorize the distribution of CYP2C19 phenotypes. More than eighty percent of the metabolism of omeprazole, lansoprazole, and pantoprazole is attributed to the enzyme CYP2C19 8, 9. The CYP2C19 is responsible for the metabolism of esomeprazole, which is the S-isomer of omeprazole, to a lesser amount than omeprazole itself. A reduction that does not include enzymes is the primary mechanism by which rabeprazole is converted into rabeprazole thioether. Because of this, the efficacy of omeprazole and lansoprazole may not always be sufficient for those who have an exceptional metabolic rate or an ultra-rapid metabolism 10, 11. Because multiple doses are necessary to inhibit freshly generated PPIs and achieve maximal acid-inhibition, the action of PPIs on stomach acid production is sluggish and reaches a plateau in three to five days. This is among the other problems associated with PPIs 12.

Vonoprazan (Takecab®), manufactured by Takeda Pharmaceutical Co. Ltd. in Tokyo, Japan, is a novel potassium-competitive acid blocker (P-CABs) that was approved for the first time in Japan in the year 2015. Vonoprazan competitively blocks the potassium-binding site of H+/K+-ATPase, and its inhibitory activity on gastric acid production is more stable than that of PPIs due to its greater pKa value 13, 14. Preclinical research investigations have demonstrated that vonoprazan accumulates at high concentrations in the cells of the gastric glands and gradually clears out. This leads to a longer and higher elevation in the stomach's pH 15. Vonoprazan powerful inhibitory effect on stomach acid formation makes it an excellent treatment for gastric acid related disease 16. Recent comparative trials on peptic ulcer management have demonstrated that vonoprazan has a more potent acid-inhibiting effect than PPIs.

It is normal practice in clinical to utilize vonoprazan and PPIs to treat diseases linked to gastric acid. Even though their primary mechanism of action is to limit the secretion of gastric acid, their influence extends to diagnostic imaging methods that include the gastrointestinal tract 17. Before endoscopic tests, these drugs are frequently given to patients to enhance vision by lowering the amount of acid that is produced by the stomach. This helps in the identification of abnormalities such as ulcers or tumors. Additionally, the fact that they affect the pH levels in the stomach and esophagus may make it more difficult to understand the results of pH monitoring investigations, particularly when it comes to evaluating gastroesophageal reflux disease 18. Moreover, modifications in gastric motility that are brought about by vonoprazan and PPIs have the potential to have an impact on gastric emptying tests 19.

Treatment of GERD with micro/nano drug delivery systems requires imaging tools for the development and evaluation 20. Using a combination of endoscopy and confocal laser endomicroscopy (CLE), researchers may examine the interaction between the mucosa and drug-loaded nanoparticles in real-time with great resolution 21. Imaging the gastrointestinal system with magnetic resonance imaging (MRI) allows for non-invasive, high-contrast imaging, which helps monitor the location and movement of nano drug carriers 22. Radiolabelled nano drug delivery systems can be monitored using functional imaging techniques like positron emission tomography (PET) and single photon emission computed tomography (SPECT), which aid in assessing the biodistribution and targeting efficacy of these systems. By monitoring the movement of fluorescently labelled micro/nano carriers in real-time, fluorescence imaging sheds light on how well drugs are absorbed by the gut 23. The combined use of these cutting-edge imaging techniques has improved our knowledge of the efficacy and therapeutic potential of micro/nano drug delivery systems for the treatment of GERD. Therefore, we provide a systematic review of PPIs and vonoprazan pharmaceutical formulations. Additionally, we extend a prospective view on the clinical imaging of the vonoprazan formulation for disorders associated with gastric acid.

## Mechanistic Approach of Potassium-Competitive Acid Blockers (PCABs)

During the process of acid secretion, the surface of the H+/K+-ATPase, also known as the proton pump, exposes itself to the very acidic parietal cell canaliculus 24. This canaliculus has a strong affinity for K+. Antibiotics that compete for the binding of potassium ions have the potential to limit acid secretion. This is because cations play an important role in enzyme activity 25. This notion serves as the foundation for the mechanism of action that P-CABs possess. Due to its capacity to block the proton pump in a rapid, efficient, and reversible manner, P-CABs were initially created in the 1980s and have been the subject of research by numerous pharmaceutical companies all over the world. Schering-Plough was one of the companies that succeeded in developing a prototype of the P-CAB, which was designated as SCH28080 26.

Although the mechanism of action was not completely understood at the time, it was discovered that this medication reduced the amount of acid that was produced by the stomach in humans 27. The ability of SCH28080 to inhibit gastric H+/K+-ATPase by competing with K+ was later proven. Due to the hepatotoxicity that was caused by repeated therapy, however, the clinical development of SCH28080 was terminated. An important distinction between these innovative antisecretory drugs and PPIs is that they compete with potassium (K+) and cause a dose-dependent, selective, and reversible blockage of the proton pump 28. Since they are not prodrugs that require activation in parietal cells similar to PPIs, they begin functioning immediately, and the control of acid production begins during the first day of treatment after the initial dose is administered. It is also possible for them to remain in the mucosa of the stomach for up to twenty-four hours 29. The acid-inhibitory impact of these medications is consistent throughout the day and night, in contrast to the effectiveness of PPIs, which are less effective throughout the night 30. There is a category of weak bases known as P-CABs. For example, the pKa value is one of the most important identifiers; the lower the pKa value, the more potent each acid is. Different drugs have different pKa values, ranging from 5.6 (SCH28080) to 6.1 (linaprazan) to 9.3 (vonoprazan). The pKa values for various pharmaceuticals are quite varied. Since vonoprazan comprises a pKa at 9.3, the bulk of it is protonated in a short amount of time and can exert its inhibitory effect through this mechanism 31. Therefore, these protonated versions of P-CABs tend to cluster in the acid-secreting canaliculi of parietal cells, where they can inhibit the enzyme that is responsible for the production of H+ and K+ ATPase 32. The reason for this is that the protonated forms have a lower probability of penetrating membranes compared to the non-ionic molecules 33. K+ competitive acid blockers inhibit acid secretion. The fact that P-CABs are capable of blocking the K+ exchange channel of the proton pump is a significant factor; they can restrict acid secretion in a manner that is both highly competitive and reversible. This is because of their ability to block the channel. On the other hand, they bind in a reversible manner rather than a covalent approach, it is essential to maintain a steady plasma concentration of the drug to deliver an effect that lasts for a longer period. They may have an effect that lasts for a longer period than the PPIs that have an extended-release formulation 34. This is entirely reliant on the drug's half-life. In the case of vonoprazan, which displayed rapid absorption and a mean elimination half-life of up to nine hours, there was no indication of a time-dependent inhibition of metabolism. Vonoprazan can be eliminated from the body quite quickly 35.

## Physicochemical characteristics and pharmacokinetic assessment of proton pump inhibitors and vonoprazan

An inhibitor of the H+/K+ ATPase enzyme in the parietal cells of the stomach, PPIs lower the generation of gastric acid. The acidic stomach environment activates these weak bases, which have a pKa of about 4-5. PPIs are lipophilic and are easily absorbed in the small intestine. Being prodrugs, they must be activated by acids in order to produce the active sulfenamide, which forms a covalent bond with the proton pump. Pantoprazole, omeprazole, and esomeprazole are the three most used PPIs 36. Although they have somewhat different pharmacokinetics, they all have comparable physicochemical characteristics. To alleviate symptoms associated with acidity, physicians use vonoprazan. The molecular weight is 345.81 g/mol, and the powder has a crystalline structure that ranges from white to slightly yellow 37. Because of its limited solubility in water and excellent stability in acidic environments, vonoprazan is useful for controlling the pH of the stomach. The compound's high affinity for the gastric H⁺/K⁺ ATPase enzyme is due in part to its basic pKa of 8.7. With a Log P value of 2.9, vonoprazan is a lipophilic, which helps it pass through biological membranes and ensures that it is effectively bioavailable when taken orally. The only way for proton pumps that are activated in reaction to food to form persistent inhibitory complexes with PPIs is through a series of stimuli, including hormones, peptides, and other transmitters 38.

FDA suggests lansoprazole and omeprazole for CYP2C19-sensitive index and it comes under BCS class II 39. The adult dose of omeprazole varies according to the disease condition for example to treat a duodenal ulcer the dose of omeprazole is 20 to 40 mg 40. However, the adult dose of lansoprazole is 15 mg to treat GERD 41. Moreover, the adult dose of vonoprazan tablet is 20 mg 42. However, following oral treatment, approximately 70% of active proton pumps can be successfully blocked because PPIs have a relatively short half-life (0.5-1.5 h, excluding tenatoprazole). Research shows that omeprazole, lansoprazole, and esomeprazole are more influenced by food consumption when taken at the same time as pantoprazole, rabeprazole, and dexlansoprazole. Symptom improvement becomes apparent after two or three days of treatment when the patient reaches steady state, with 66% inhibition of stomach acid output. When PPIs are used twice a day, the maximum secretory inhibition is increased to 80% 43. However, due to their short half-life, subsequent administration of PPIs does not provide substantial relief in cases of acid reflux that occurs during the night. With a half-life of up to 9 hours, tenatoprazole is the only exception to this rule. Even after discontinuing treatment, this medication effectively reduces acid secretion throughout the nocturnal. At daytime, though, its inhibitory activity falls short of expectations. The maximum concentration (Cmax) and the area under the curve (AUC) are utilised to characterise the effectiveness of PPI inhibitory action 44. The drug's efficacy is proportional to the amount of time the plasma level remains over this threshold. Protein-protein interactions are defined by their strong protein binding and high bioavailability. Many liver enzymes break them down, but the CYP219 isoenzyme is particularly important.

An acid blocker and PPIs that compete for potassium was studied in terms of its pharmacodynamics and pharmacokinetics in a study conducted in the United States. Healthy adults were randomly assigned to either a 7-day washout period between courses of lansoprazole 30 mg once daily and vonoprazan 20 mg once daily, or the other way around. On day 1 (62.4% vs 22.6%, P < 0.0001) and day 7 (87.8% vs 42.3%, P < 0.0001), the proportion of 24-hour periods with a stomach pH greater than 4 was higher for vonoprazan compared to lansoprazole. Differences in pH began to manifest 2.5 hours after the initial dosage. Compared to lansoprazole, vonoprazan effectively reduced intragastric acidity more quickly and with higher strength in US individuals 45.

Twenty healthy adult male volunteers with CYP2C19 extensive metaboliser genotype were studied in another study by Sakurai *et al.* They compared the acid-inhibitory effects of vonoprazan to those of two control PPIs, esomeprazole and rabeprazole, and measured how quickly and for how long these effects wore off. The subjects in this two-period cross-over trial were given 20 mg of vonoprazan or 10 mg of rabeprazole or 20 mg of esomeprazole orally once a day for seven days. The main pharmacodynamic endpoint was the average gastric pH and the percentage of time that pH was greater than or equal to 3, 4, or 5 (pH holding time ratios, or HTRs). On both days, vonoprazan had a significantly higher acid-inhibitory effect (pH4 HTR) than esomeprazole or rabeprazole. On day 7, the difference in pH4 HTR between vonoprazan and esomeprazole was 24.6%, and between vonoprazan and rabeprazole, it was 28.8%. Compared to esomeprazole and rabeprazole, vonoprazan had a 24-hour pH4 HTR ratio greater than 0.8 from day 1 to day 7. In most cases, vonoprazan had no ill effects. A rash that cleared up after discontinuation caused one vonoprazan patient to withdraw from the study. Compared to esomeprazole (20 mg) and rabeprazole (10 mg), vonoprazan (20 mg) had a stronger and longer-lasting acid-inhibitory effect in this investigation. Hence, vonoprazan could provide a novel approach to treating acid-related disorders 46.

## Comparison of the pharmacological effects of proton pump inhibitors and vonoprazan

The maximum plasma concentration (Cmax) of vonoprazan can rise from 10 to 60 ng/mL in just 1.5-2 hours, and it is acid-stable, making it an ideal candidate for fast-release therapy. Additionally, it is dramatically affected by intestinal meal absorption and has an area under the curve (AUC) from zero to infinity in a dose range of 1.14-1.32 ng.h/mL 47. Vonoprazan outperforms PPI in terms of the beneficial Cmax, AUC, and half-life, but there is no discernible difference in the holding time ratio at pH > 4 or the time required to reach Cmax. Because it has more positive charged points and is more abundant in the secretory canaliculi of stomach parietal cells than in plasma, its negative logarithm of acid dissociation constant (pKa) > 9.0. Alpha-1 acid glycoprotein and albumin determine its distribution. There is no need to activate vonoprazan with acid unlike PPI 48. Metabolised in part by SULT2A1, cytochrome P450 2C19, cytochrome P450 2D6, and cytochrome P450 3A4 (CYP3A4) in the liver. Due to clarithromycin's potent CYP3A4 inhibitory effects, which decrease vonoprazan metabolism, the pharmacokinetic interaction between the two is synergic. The converse is true for prodrug activation and PPI efficacies; CYP2C19 is the principal metabolizer of PPI and has a large metabolizer polymorphism. After the important role of H+/K+-ATPase in the last step of gastric acid secretion was discovered, research on acid suppression drugs took a dramatic turn. Disulfide linkages are formed between the cysteine component of H+/K+-ATPase and PPI, a prodrug that is activated by acid 49. After three to five days of treatment, PPI achieves its maximum acid stability. Alternative acid-suppressing drugs are being studied because PPI fails to establish a gastric basic environment. Alternately, the effectiveness of H+/K+-ATPase could be limited by lowering the concentration of potassium ions. By interacting with H+/K+-ATPase, P-CAB drugs like vonoprazan reversibly decrease the activity of potassium ions. In the acidic environment of the gastric secretory canaliculi, vonoprazan binds non-covalently to H+/K+-ATPase. The gastric pH rises to about 7 in about 4 hours due to vonoprazan slow dissociation and long-term suppression of H+/K+-ATPase production. Instead of PPIs, vonoprazan may be useful in treating gastroduodenal ulcers and GERD 50. The results of using PPIs as a treatment for these disorders are not up to par. To alleviate symptoms of erosive esophagitis rapidly and effectively, PPIs can be used in place of vonoprazan.

## Pharmaceutical formulations for gastric acid related disorders

Several different distribution modalities are included in pharmaceutical formulations of PPIs and vonoprazan to cater to the various requirements of patients (Table.1). These formulations often consist of oral tablets, capsules, and they are frequently designed to have a delayed-release formulation, and nanoparticles. This formulation ensures that the medication is effective by releasing it in the intestines rather than in the stomach. Additionally, oral suspensions provide an alternative for individuals who have issues swallowing in addition to this 51. Formulations that are administered via injection have an important role in therapeutic settings, particularly for patients who are hospitalized and have serious illnesses. Some PPIs are also used in combination therapy, such as when they are used with antibiotics to treat Helicobacter pylori 52. Additionally, there are treatments available for frequent heartburn that can be purchased without a prescription 53. The patient needs to adhere to the prescribed dosages and formulations to achieve the best possible treatment outcomes.

### Delayed-Release Tablets

A delayed-release pill is the most common form of administration for PPIs. Enteric polymers must coat the tablet to prevent the API from deteriorating. Polymers derived from methacrylate, such as a copolymer of methacrylic acid and ethyl acrylate, are the most popular 69. For several acid-related conditions, PPIs are highly effective. Nevertheless, PPIs must be shielded from the acidic stomach environment when taken orally due to their acid-labile nature 70, 71. These delayed-release (DR) formulations are hampered by the many different kinds of enteric coatings that have been developed to protect the PPIs. To dissolve and be absorbed, DR-PPIs must first reach the stomach and have their enteric covering removed. The proximal small intestine is the site of most PPIs absorption. After absorption, PPIs travel throughout the bloodstream, with parietal cells being the most selective recipients, especially during acid secretion 72. The parietal cell is responsible for excreting PPIs into the canalicular space through its luminal surface 73. Following the process of protonation and transformation into a sulfenamide derivative, the activated PPIs molecule establishes a covalent link with the cysteine moieties of the H+, K+-ATPase molecule that is bound to the membrane environment. This occurs through the process of protein-protein interaction. No significant differences in antisecretory activity or clinical consequences have been definitively linked to the fact that PPIs differ in the cysteine residues that they bind 74.

In a study, Rukari *et al.*, developed delayed release tablets of Esomeprazole magnesium for gastric disease. In contrast to immediate-release dose forms, delayed-release dosage forms allow the medicine to be released from the container at a later time. Esomeprazole concentrations in pharmaceutical formulations can be estimated using a UV spectrophotometric technique. Due to the drug's cohesive characteristics, the tablets were manufactured using the wet granulation process instead of direct compression. Then, to protect the optimized core tablet from moisture, it was enteric coated using a base coat polymer cellulose derivative. To find the best coating, they compared it to a commercially available sample (ESOZ). The release of Esomeprazole from enteric-coated tablet dissolving studies was observed in a pH 1.2 simulated gastrointestinal fluid, with the majority of the drug being released in a pH 6.8 phosphate buffer. When compared to the marketed sample dissolving profile, the developed formulation ultimately produced satisfactory results. This means that the final formulation can be utilized as a single unit dosage to treat acid-related disorders without Esomeprazole degrading due to the enteric coating technique. As a result, a strong, pharmaceutically-compatible version of the delayed-release pill Esomeprazole was created 55.

### Delayed-Release Capsules

Polymers that are known as pH-dependent and gel-forming polymers, or a combination of the two, are made available by pharmaceutical technology for use in enteric coating and the fabrication of pharmaceutical dosage forms through the utilization of Enteric Capsule Drug Delivery Technology (ECDDT) 75, 76. Because of their acidic composition, these polymers hinder the drug's solubility in the acidic stomach environment but enable its release in the small intestine's neutral environment 77, 78. Acetate succinates, phthalates, trimellitates, or acidic poly-(meth)acrylate copolymers are typical examples of these compounds. Other popular examples include cellulose and vinyl derivatives. At pH 5-7, they typically disintegrate. To make ECDDTs, these polymers are mixed with HPMC, which has better qualities than gelatine, to create the capsule cap and body shell 79.

**Figure 1:**
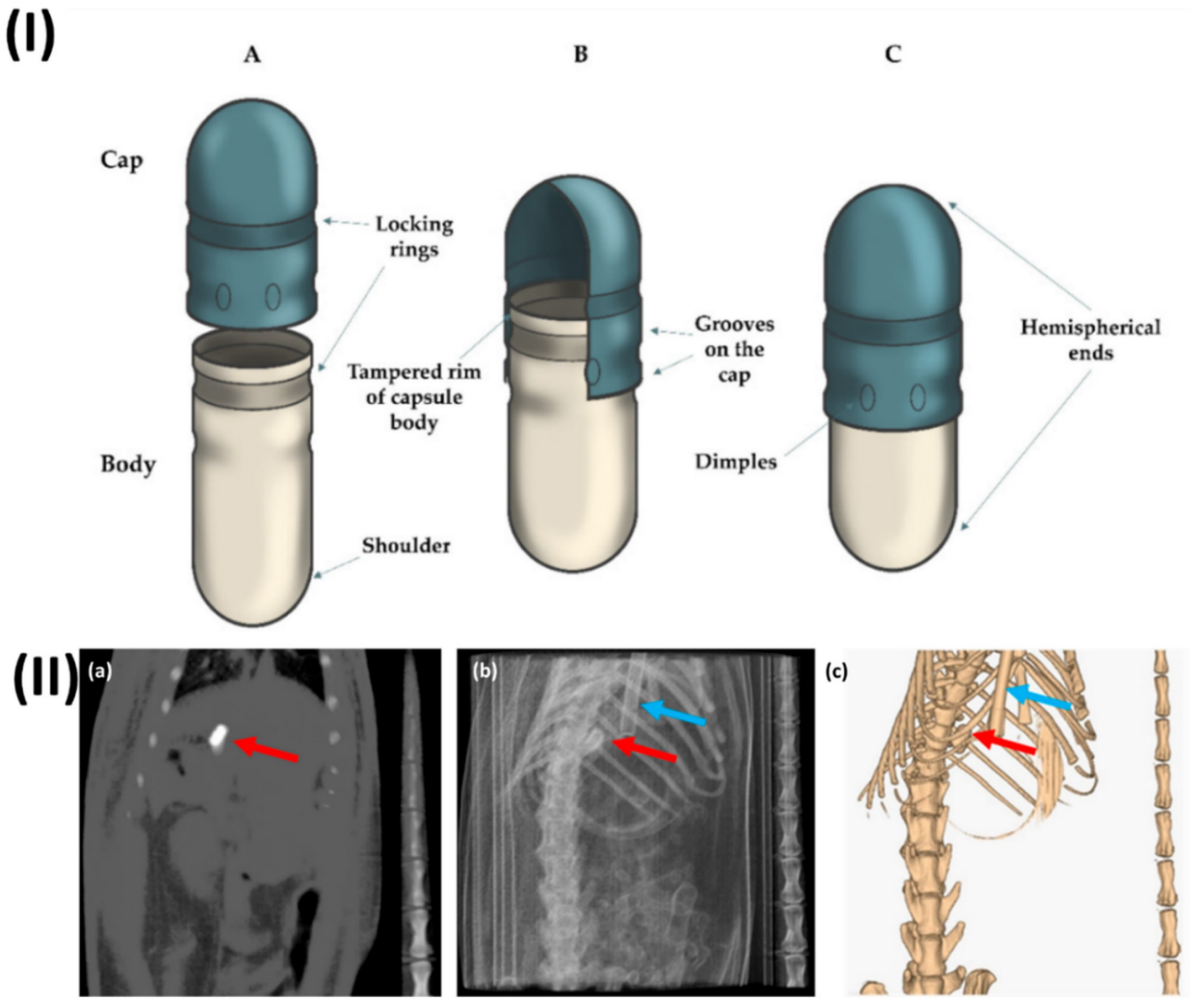
(I) A typical type of enteric hard capsule that is made with ECDDT and its respective structure (**A**) both the cap and the body of the capsule is in an open state.; (**B**) position that is pre-closed; (**C**) hard capsule in its closed configuration. Reproduced with the permission from ref 79. Fig 1 (MDPI©2022). (II) (**a**) CT slice of the size 9h capsule that was taken from the stomach of the rat, (**b**) projection of the planar CT, and (**c**) an image of the same animal produced in three dimensions using CT. There is a red arrow that points to the capsule, and there is a blue arrow that points to the breathing sensor. Reproduced with the permission from ref 80. Fig 3 (MDPI)©2020).

In a study, Lado *et al.* examined the stomach emptying rate of coated capsules in rats. The capsules were followed and located using computed tomography (CT) imaging as they travelled through the intestines. A contrast agent called barium sulfate was placed into two commercially available mini-capsules made of gelatine (size 9 and 9h) and then coated with Eudragit L. While under the influence of anaesthesia, not a single capsule passed through the digestive tract. Most size 9 capsules failed to empty from the stomach in rats that had not been anesthetized, while smaller size 9h capsules did empty and made it into the intestines. This study shows that solid oral dose forms still can't be guaranteed to empty from the stomach, even when tested on rats. Furthermore, both capsule sizes were discovered to have their stomach emptying stopped when anaesthesia was administered. In this study, they provide more evidence that CT imaging can be used to successfully visualize and localize solid dose forms in rats' intestines without the need for anaesthesia (Fig. 1) 80.

### Minitablets

Mini-tablets are often smaller than regular tablets, typically having a diameter of less than 2.0 mm. One benefit is that there is little mass variation due to their consistent size 81, 82. As with other multiple-unit dosage forms, they are simple to make; they may be compressed using standard tablet presses with specially developed multi-tip punches, coated to alter drug release, and put into capsules 83. Also, mini-tablets are highly dispersible in the digestive tract, which reduces the likelihood of high local drug concentrations, and they have great production repeatability, which means there's less chance of dose dumping and bioavailability fluctuation. Consequently, mini-tablets can serve as a suitable substitute for granules, pellets, and other MUPS. Lastly, oral disintegrating, gastroretentive, bioadhesive, and paediatric dosage forms can all benefit from improved release methods that increase drug bioavailability 84.

**Figure 2:**
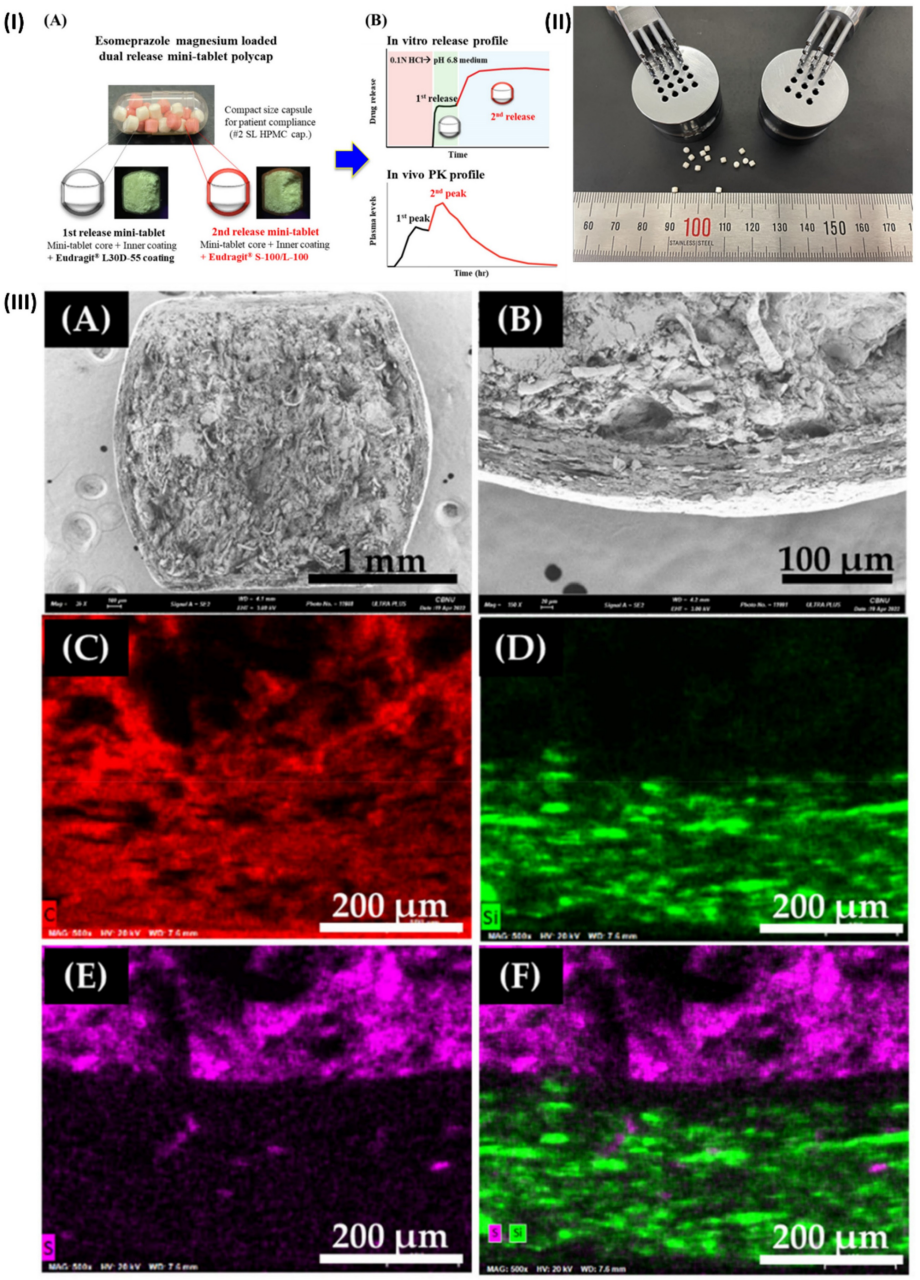
(I) Minitablets of esomeprazole magnesium (DR polycaps). (**A**) photo of DR polycaps images. (**B**) Pharmacokinetics profiles of DR polycaps (II) Multi-tip punches and a mini-tablet measuring 2 millimetres in size (III) The coated EMP mini-tablet was mapped using SEM and then imaged using SEM-EDS. Reproduced with the permission from ref 57. Fig 1, Fig. 2, and Fig. 5 (MDPI©2022).

In a study, Kwon *et al.* developed a new DR polycap containing esomeprazole magnesium to prevent NAB, with an extended onset time and enhanced bioavailability. Esomeprazole magnesium (EPM) mini-tablets ensured speedy medication release because of their innovative formulation. The first-release mini-tablet was prepared by covering the core with an inner coating and an aqueous dispersion coating of Eudragit® L30D-55. The *in vitro* dissolution test and microscopic analysis were used to characterize each variety of mini-tablet. To create DR polycaps, ten of each mini-tablet was combined with hard capsules after testing. The optimal combination of mini-tablets was determined through *in vivo* pharmacokinetic investigations and *in vitro* release testing. While the DR polycap had an AUC0-24h that was comparable to a commercial medication (Nexium®), it had a Cmax that was around 50% lower and an extended Tmax that was around 1.7 times longer. To conclude, DR polycap's dual-release properties make it a viable substitute for existing medications that enhance NAB and dosage compliance (Fig. 2) 57.

### Pellets

Granules that are 0.5-1.5 mm in size and have a spherical form are called pellets. They have multiple potential dosage forms, including oral suspension, multi-particulate tablets, and capsules or sachets 85. There are two main ways to make pellets: either coating inert cores with the active ingredient or creating pellets from the bulk that already contains the active ingredient (using the extrusion-spheronization or granulation process). Subsequent functional layers can be applied to both of the strategies 86. The capacity of pellets to offer regulated and prolonged drug release makes them an important dosage form in pharmaceutical manufacture. For pellets to be consistent, effective, and stable, their production must adhere to several strict guidelines. Initially, the powders are mixed and ground into small, homogeneous pieces 87. Wet granulation involves adding a binding solution to the powder, while dry granulation uses pressure to compact the powder. Both procedures are used for granulation. Pellets are formed from the granulated material using processes such as extrusion-spheronization or pan granulation 88. In the former, the granules are extruded through a die and subsequently spheronized into round pellets. In the latter, the powder is agglomerated into pellets in a revolving drum. To keep the pellets stable and stop them from degrading, it is essential to dry them after they are formed. To alter the medication release profile or shield the pellets from outside influences, coating is frequently used. Film coating and enteric coating are two methods that can do this. The pellets' size, homogeneity, and drug release properties are rigorously controlled throughout the manufacturing process to guarantee they satisfy the specifications. Pellets controlled release characteristics contribute to better treatment results and higher patient compliance 89.

**Figure 3:**
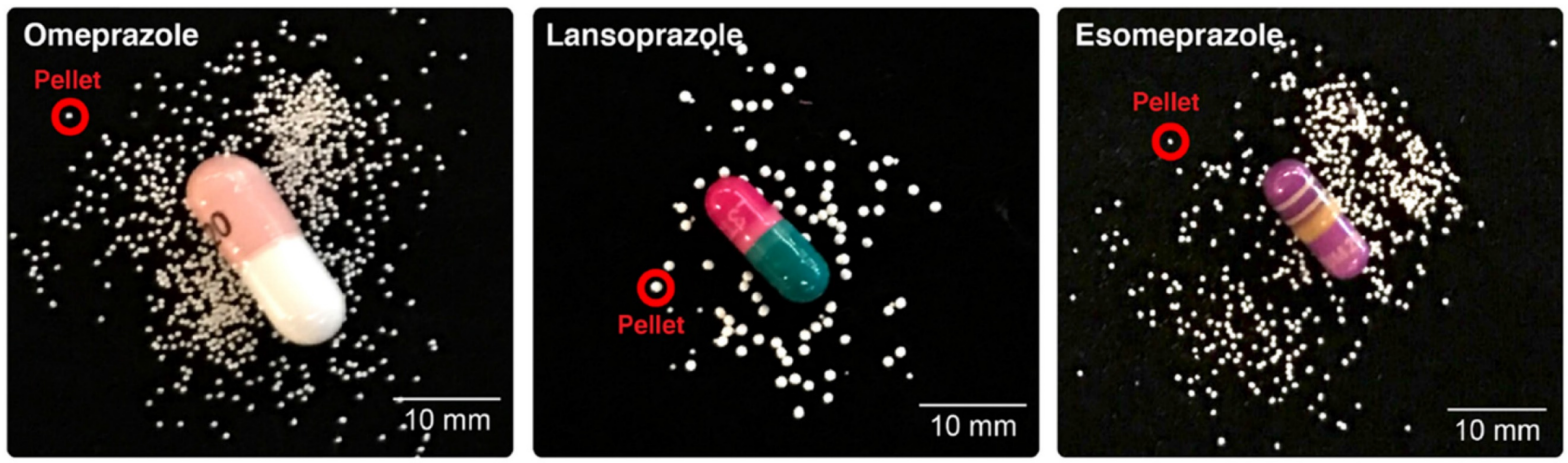
To determine the effectiveness of each medication, three distinct controlled-release pellets, similar to the ones that are circled in red, were extracted from capsules and evaluated at each pH level. Reproduced with the permission from ref 90. Fig.2 (Springer Nature©2020).

In a study, Bhakta *et al.* have demonstrated a straightforward and entirely automated method for extracting profiles of dissolution from individual pellets. Rather than tracking the rise in drug compound concentration during dissolution, which is the conventional approach. The mass of the glass tube and its contents inversely determines the resonance frequency, which an electrical circuit maintains. As a buoyant mass changes about the flow of a pellet through the tube, there is a transient shift in the resonance frequency. With high mass resolution (700 nanograms) and high temporal resolution, they can monitor the degradation mass by passing it through the vibrating sensor tube. To demonstrate the feasibility of this method, they first compared brand-name and generic versions of the same medicine and then measured the single-pellet dissolving characteristics of various commercial controlled-release PPIs in simulated gastric and intestinal contents. Dissolving profiles for the various medications were shown to be significantly different based on the data collected from vibrating tube sensors. In certain instances, their method also showed variances between different pellets of the same pharmacological product. It can supplement or even replace current dissolution tests, which helps with pharmaceutical product development and quality assurance (Fig. 3.) 90.

### Fixed-Dose Combination Products

For specific quantities, a single dosage form containing two or more active substances is called a fixed-dose combination (FDC) 91. Most FDC medications are designed to treat a specific illness; for example, antiviral FDCs like Truvada® and Atripla® treat HIV with tenofovir and emtricitabine, respectively 92. FDCs can also target multiple diseases at once; for example, Juvisync^TM^ (sitagliptin and simvastatin) treats both type 2 diabetes and high cholesterol. The following reasons contribute to the rising popularity of FDCs as treatment options: (a) improved patient compliance and adherence in conditions like diabetes (1) and hypertension (2); (b) more ease and convenience for patients and less complexity in managing conditions like AIDS and cardiovascular diseases; (c) lower costs for patients (one co-pay); and, finally, (d) FDC products offer a great chance for products to be managed throughout their life cycle 93. Combination drug products offer several benefits over single-active pharmaceuticals, including the potential for lower dosages of each active ingredient, fewer side effects, and increased clinical efficacy as a result of the additive or synergistic effect of each active ingredient. It is important to carefully examine the active ingredients' action processes before deciding to combine them. Choosing compounds that target distinct pathways is crucial for effective treatment, as there might be multiple biological pathways involved in disease progression 91. This will decrease the chances of drug resistance and clinical relapse. So, to make sure that FDC products are helpful in disease management and safe for patients, it's important to carefully choose which medications to combine. Many rules and regulations about the creation of FDC products have been laid out by regulatory bodies 93. More information on these can be found in the section titled "regulatory requirements for bioequivalence of FDC. As a general rule, while trying to get an oral FDC product registered, one must demonstrate that it is bioequivalent to taking the monotherapy drugs alongside the FDC 94.

### Bilayer Tablets

The concept of bilayer is utilized by the Skye Pharma PLC's in their Geomatrix tablet. With this method, it is possible to combine many medications into a single dose. By combining layers of various polymers, it is possible to control the release rate of more than one drug at once 95. This opens up new possibilities for drug delivery, such as targeted drug delivery in the gastrointestinal tract using polymers that depend on pH or a bolus release followed by controlled release. Although the drug release is not controlled by the mechanical strength of stacked tablets, knowing this attribute could help with understanding the adhesion between layers and better characterizing the systems 96. The first layer of a bi-layer tablet releases the medicine immediately, while the second layer releases the drug at a later time, either as a second dosage or throughout a longer release period. Two compounds that are incompatible with each other can be separated using a bi-layer tablet, and a sustained-release tablet with an immediate-release layer for the first dose and a maintenance-dose layer can be used for a variety of purposes 97. To develop controlled release tablet formulations with surrounding or several swelling layers, as well as systems for the administration of incompatible medications, multi-layer tablet preparations are utilized. Compressing the several geometries given here will necessitate a wide range of approaches 98. The benefits of bilayer tablets over their monolayer counterparts are substantial. To physically separate chemically incompatible components of a formulation, such tablets are often utilized. Combining layers with different release patterns or layers that release slowly and layers that release quickly has allowed for the development of bilayer tablets, which in turn have permitted the controlled distribution of active medicinal components with pre-determined release profiles 99.

**Figure 4:**
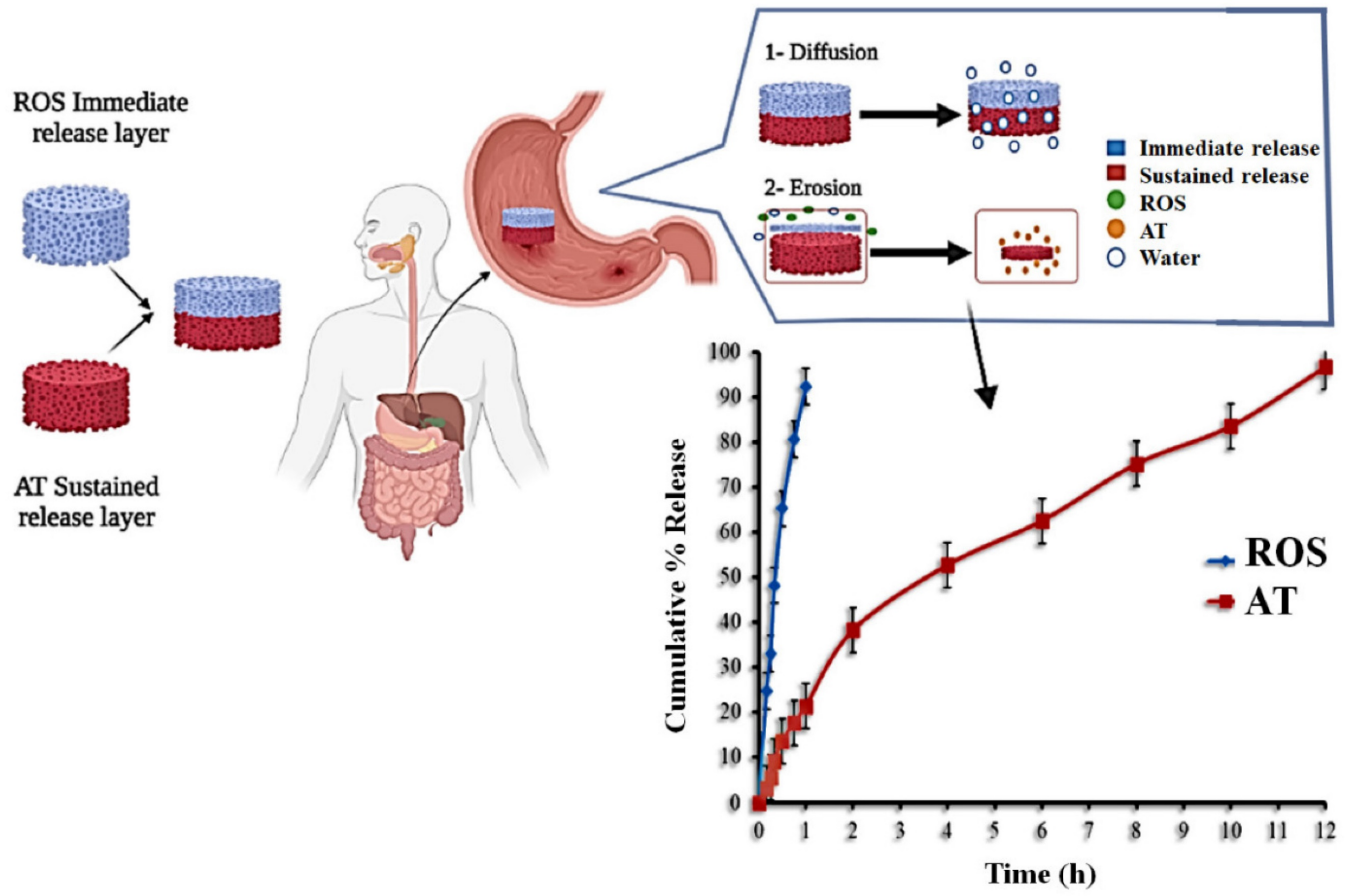
Schematic representation of the ROS/AT bilayer floating tablet. Reproduced with the permission from ref 100. Figure 1 (MDPI©2022).

Elsayed *et al.*, developed bilayer tablet of rosuvastatin calcium (ROS) and atenolol (AT) for the treatment of dyslipidemia and hypertension. The immediate-release layer made use of a solid ROS dispersion with sorbitol at a ratio of 1:3 w/w, while the floating sustained-release layer included HPMC, EC, and sodium bicarbonate. To maintain AT release, 3^2^ full factorial designs were used to adjust the concentrations of HPMC and EC. The bilayer pills were made using the compression technique that is directly compressed. At a pH of 1.2, 92.34 ± 2.27% of ROS was released within 60 minutes, according to the immediate-release layer. Under the same settings, the second layer of the bilayer tablets that released AT slowly (96.65 ± 3.36% within 12 hours) was observed. It was discovered that the non-Fickian diffusion model (zero-order, Higuchi) governed the release of reactive oxygen species from the produced tablets, while the Korsmeyer-Peppas model governed the release of ATP. The effects of ROS/AT pills on lipid profiles and blood pressure were studied in preclinical trials with rabbit models. Rabbits were made to gain weight by feeding them a high-fat diet. Comparing the bilayer ROS/AT pill group to the control group, we find that they significantly reduced lipid profiles, slowed weight growth, and maintained normal blood pressure (Fig. 4) 100.

Nirmal *et al.*, formulated a bilayer tablet of atorvastatin calcium (AT) and nicotinic acid (NA) as an extended release layer. Super disintegrant croscarmellose sodium was used to prepare the immediate release layer, whereas hydroxypropyl methyl cellulose (HPMC K100M) was used to prepare the prolonged release layer. The HPLC method was used to estimate the amounts of AT and NA released at various time intervals. The bilayer tablets exhibited no notable alteration in their appearance, drug content, or dissolving pattern over three months of storage at 40 °C with 75% relative humidity (RH). Analysis using thermogravimetry and differential thermal analysis (TG/DTA) made it very clear that the polymer composition affected the medication release from the tablet. Based on the findings, bilayer tablets may be a good way to administer AT and NA 101.

### Mucoadhesive delivery systems

The controlled release of drugs can be improved when mucoadhesive properties are coupled to tablets. This is because the drugs are more efficiently absorbed and their bioavailability is increased because the tablet has a high surface to volume ratio, which allows it to come into much closer contact with the mucus layer 102. The potential for both localized and systemic controlled release of medications is offered by mucoadhesive tablets, which can be modified to stick to any mucosal tissue, including the stomach 103. Localized drug delivery is achieved by applying mucoadhesive tablets to the gastric epithelium's mucosal tissues 104.

In a study, Alhakamy *et al.*, formulated a mucoadhesive polysaccharide polymer and prepared mucoadhesive tablets of repaglinide. The ester formation process between the hydroxyl group of xanthan gum and the carboxyl group of thioglycolic acid was used to thiolate the gum. By utilizing a central composite design, the synthesis of TXG was optimized. FTIR, rheology, and Ellman's assay (to determine the quantity of thiol/disulfide groups) were utilized to conduct further studies on TXG and STX. The enhanced interactions of macromolecules responsible for increasing the mucosal adhesion strength of thiolated gum may explain why STX's viscosity increases significantly. Cytotoxic research data supports the safety of STX. Both the *ex vivo* mucoadhesion strength and the residence length (>16 h) were highest for mucoadhesive formulations of STX that contained repaglinide. Repaglinide was controlled and released over 16 hours in the thiolate and S-protected thiolate formulations due to the matrix's increased cross-linkage and cohesive nature. The RSX-2 formulation had a longer half-life and a larger area under the curve (AUC), according to the pharmacokinetic analysis. This suggests that drug systems based on S-protected thiomers can be useful for increasing the bioavailability of medicines with limited solubility 105.

Moreover, Hou *et al.*, developed chitosan alginate microparticles for gastroretentive delivery. A mixture of cationic components (chitosan and Ca2+) and anions (alginate) were used in an emulsification-internal gelatine process to generate the microparticles. Puerarin mucoadhesive microparticles, when administered 150 mg/kg, 300 mg/kg, 450 mg/kg, and 600 mg/kg body weight before ethanol consumption, considerably protected stomach ulceration in ethanol-induced gastric ulcers.

When comparing stomach tissues from the experimental group with and without ethanol, notable alterations were noted in inflammatory cytokines including prostaglandin E2 (PGE2), tumour necrosis factor (TNF-α), interleukin 6 (IL-6), and interleukin 1β (IL-1β). Finally, puerarin-loaded core-shell type pH-sensitive mucoadhesive microparticles may improve the absorption of puerarin and help with ethanol-induced stomach ulcers 106.

### Nanoparticles

The distinct characteristics and possible uses of nanoparticles in targeted medication delivery and protective coatings make them attractive candidates for the treatment of disorders associated with stomach acid 107. One usage of nanoparticles is the encapsulation of medications for the treatment of stomach ulcers and acid reflux 108. These microscopic particles can encase medications, preventing them from being hydrolysed by gastric acid and facilitating their intact delivery to the gastrointestinal tract site of action. When compared to more traditional drug formulations, this targeted delivery system results in lower dosage requirements and fewer adverse effects 109. Second, functionalized nanoparticles can attach to the stomach mucosal lining and create a protective barrier. This barrier can protect the sensitive tissues from the harmful effects of stomach acid, which can be helpful in cases where acid erosion is a worry. Nanoparticles have the added benefit of being able to release medications in a regulated manner, which can prolong their therapeutic efficacy and improve patient compliance 110. Researchers can optimise therapy effects by modifying nanoparticles' size, surface properties, and composition 111.

**Figure 5:**
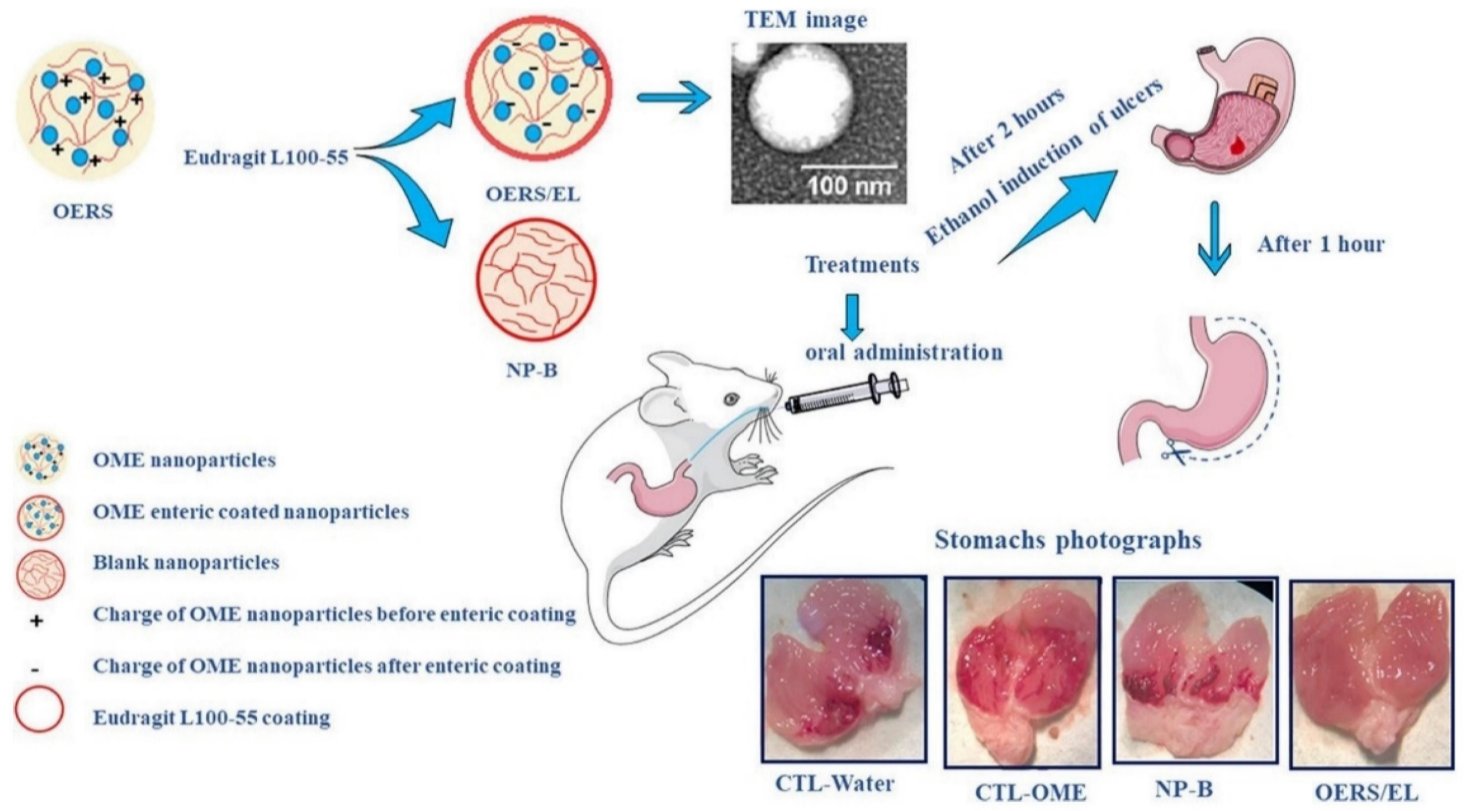
Schematic illustration of the omeprazole nanoparticles for oral administration. Reproduced with the permission form ref.^112^. Graphical abstract (Elsevier©2020).

In a study, Diefenthaeler *et al.*, developed omeprazole nanoparticles for paediatric administration. The ultimate goal of this research is to create a liquid pharmacological dosage form of omeprazole loaded nanoparticles. The nanoparticles were created using the nanoprecipitation process. The inner core was made of Eudragit® RS100, while the outside coating was made of pH-sensitive Eudragit® L100-55. To assess the pharmacological activity, ethanol ulcers were induced in mice. The average diameter of the omeprazole nanoparticles was 174 nm (± 17), their polydispersity index was 0.229 (± 0.01), their zeta potential values were -13 mV (± 2.60), and their encapsulation efficiency was 68.1%. A pharmacological evaluation in living organisms demonstrated that nanoparticles could prevent gastric ulcers in mice (Fig. 5) 112.

## Proton Pump Inhibitors and Vonoprazan Imaging Modalities for Gastric Acid Related Disorders

Gastric acid-related illnesses, particularly those treated with drugs like vonoprazan, rely heavily on imaging techniques for diagnosis and therapy 113. As a first line of defense, upper endoscopy provides direct visuals of the stomach and esophagus, which can help detect inflammation, ulcers, and other anomalies 114. In addition to X-ray imaging, barium swallows and upper gastrointestinal series can provide light on structural abnormalities such as strictures or hiatal hernias. Magnetic resonance imaging (MRI) and computed tomography (CT) scans provide precise images of the abdomen, which can help detect complications such as perforation or the formation of abscesses 115. To help evaluate the degree of the condition and identify problems like tumors, endoscopic ultrasonography (EUS) produces high-resolution pictures of the layers of the digestive tract 116. Timely diagnosis and appropriate treatment strategies can be achieved by integrating various imaging modalities with clinical results to create a complete approach to the management of disorders linked to gastric acid 117.

### High resolution manometry (HRM) imaging for gastric acid related disorders

As a cutting-edge diagnostic tool, high-resolution manometry (HRM) evaluates diseases related to esophageal motility. In contrast to traditional manometry, which relies on data collected from a small number of sensors distributed along a catheter, HRM employs a network of closely spaced sensors to assess esophageal function in greater depth and breadth. HRM entails lining the esophagus with a thin, flexible catheter that is fitted with many pressure sensors 118. As a patient swallows, these sensors detect the force applied by the muscles lining the esophagus wall. The sensors are positioned so that they span the whole length of the esophagus once the catheter is introduced through the nose. To get pressure data during an HRM procedure, the patient is instructed to swallow several times. That way, physicians may check how well the lower esophageal sphincter, a muscle valve that divides the stomach from the esophagus, is working and how well the esophageal contractions are coordinated 119. The function of the lower esophageal smooth muscle and peristalsis, the coordinated wave-like contractions that move food through the esophagus, are revealed by the high-resolution data obtained from HRM. Achalasia, esophageal spasm, and inefficient esophageal motility are among the several esophageal motility problems that can be diagnosed with this thorough evaluation. HRM imaging enables accurate localization of esophageal anomalies and can direct treatment decisions, including the selection of suitable endoscopic or surgical procedures 120. To further improve the assessment of esophageal function, HRM can be used with additional diagnostic tools like impedance monitoring. Esophageal motility abnormalities can be better understood and treated with the use of high-resolution manometry imaging, which provides specific information that can be used to customize treatment plans for each patient.

**Figure 6:**
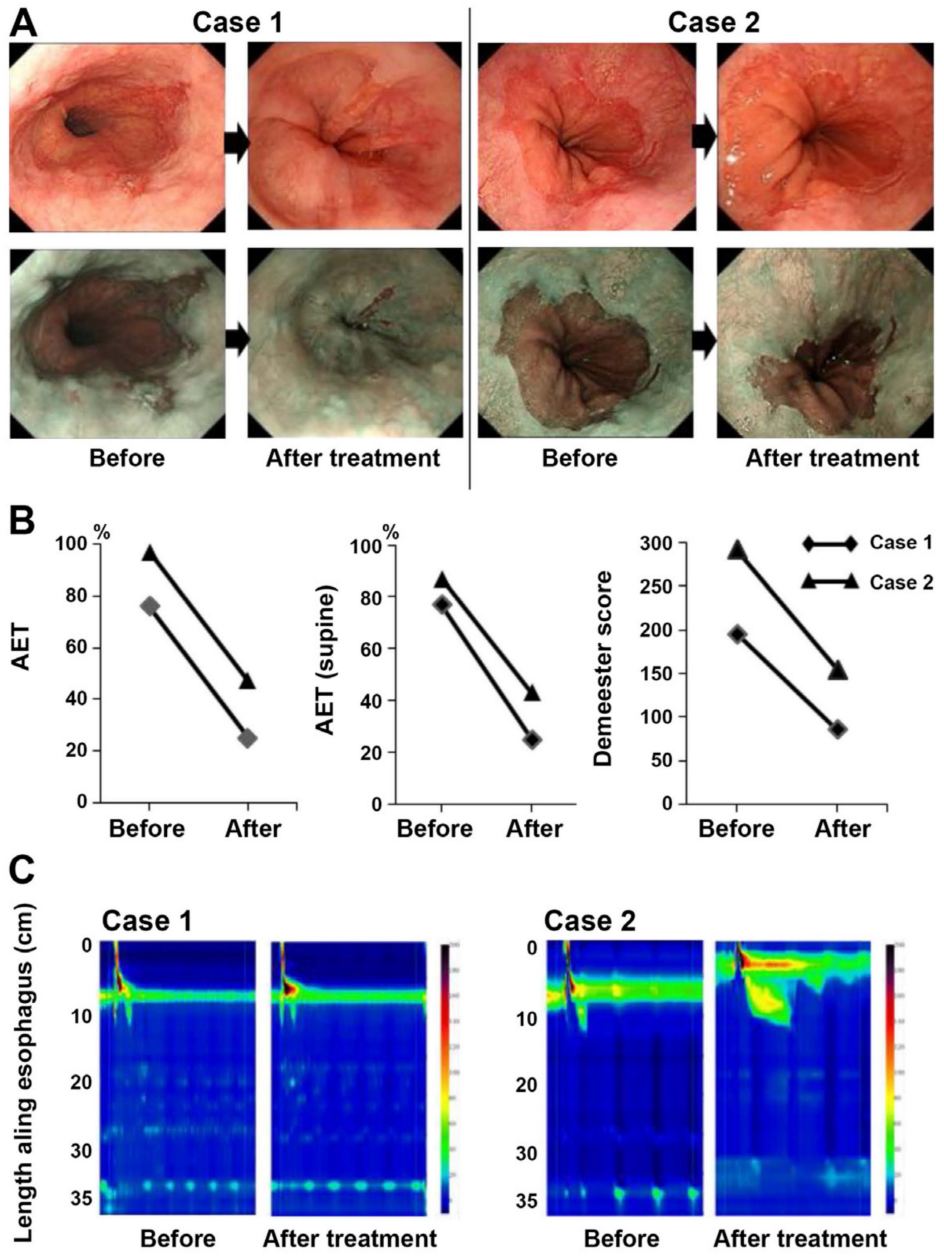
The findings of the endoscopic examination, the 24-hour pH monitoring, and the HRM. (A) White light and narrow band imaging were used to examine the endoscopic alterations that occurred before and after the vonoprazan treatment. (B) Analysis of the individual pH monitoring data at baseline and after vonoprazan administration for a period of twenty-four hours. (C) After therapy, HRM imaging with pressure topography plots was performed at the beginning of the study. Reproduced with the permission from ref 121. Fig.4 (Spandidos Publications©2021).

In a study, Tabuchi *et al.*, investigated the effects of vonoprazan in SSc‑related GERD. Before and after receiving vonoprazan treatment, 15 SSc patients with GERD had their frequency scale for symptoms of GERD (FSSG) scores recorded. Furthermore, a small number of patients underwent endoscopic esophagogastroduodenoscopy. Thirteen out of fifteen patients, or 93%, had already taken either conventional PPIs or histamine 2 receptor antagonists. The average overall FSSG score before vonoprazan treatment was significantly high (25.2±10.7) compared to a normal score of less than 8, even though most patients did not have severe erosion found during the baseline esophago-gastroduodenoscopy test. Following the administration of vonoprazan, the FSSG score dropped to 9.6±7.0. Six0.8±21.2%, 67.3±24.8%, and 55.4±26.0% were the average improvement rates of the total FSSG, acid reflux, and dysmotility scores, respectively. These findings provide more evidence that vonoprazan can alleviate GERD symptoms in SSc patients. At baseline and 12 weeks into the vonoprazan treatment, two instances had 24-hour pH monitoring and HRM recorded. Both instances showed signs of minor erosions on endoscopic inspection, and the total FSSG scores dropped from 30 to 7 and 9 to 4 respectively, after treatment. Both patients had an improvement in endoscopic findings after undergoing 20 mg of vonoprazan daily (Fig 6A). The two patients, with pH levels measured every 24 hours, showed signs of severe reflux and extreme acidosis (76 and 97%, respectively). Significantly, both the baseline Demeester scores and the supine position AET were high (77 and 87%, respectively) (Fig 6B). Following therapy, there was a trend toward slight improvement in the AET and Demeester scores (Fig 6B). Hypertension LES was found to be hypotensive (3.8 mmHg) and contractility was non-existent (3.6 mmHg; Fig 6C), according to HRM 121.

In another study, Hoshikawa *et al.* sought to find characteristics that would predict the occurrence of behavioral disorders in patients with VPZ-refractory reflux symptoms. From January 2015 to April 2020, patients at our hospital who were treated with 20 mg of VPZ for reflux symptoms (heartburn or regurgitation) but still didn't get relief underwent esophago gastro duodeno grams, high-resolution manometry, and 24-hour multiluminal impedance pH-metry (MIIpH). Individuals were categorized into the following groups: NERD, functional heartburn, reflux hypersensitivity, excessive SGB (13 per day), and potential RS according to MIIpH criteria. Final Product In a sample of 49 patients, 12.2% had SGB, 8.2% potential RS, 59.2% FH, 18.4% RH, and 2% NERD. Postprandial nonacid reflux events were more common in patients with possible RS compared to FH patients. No statistically significant predictive factors were found in the multivariate logistic regression study. In summary, behavioral issues were present in almost 20% of patients whose GERD symptoms were VPZ-refractory. Clinically, HRM and MIIpH might help with diagnosis and therapy precision 122.

### Computed tomography (CT) imaging for gastric acid related disorders

A high-tech medical imaging method for producing precise cross-sectional pictures of the body, CT imaging is sometimes called CT scanning or CAT scanning. It uses X-rays and computer technology to create extremely detailed pictures of the inside of the body, including the skeleton, organs, blood arteries, and tissues 123. The patient lies on a table that slides inside the CT scanner, which is shaped like a big doughnut, during the scan. The table is scanned using X-ray beams that are emitted from various angles all over the body. The computer uses the data collected by the scanner's detectors to generate cross-sectional pictures, or "slices," of the body by measuring the quantity of radiation that travels through it. When compared to other imaging modalities, CT scans have many benefits 124. Its superior imaging capability compared to traditional X-rays makes it an invaluable tool for the detection and diagnosis of numerous medical issues, such as cancers, infections, internal bleeding, fractures, and malignancies 125. Because of how fast CT scans can be done, they are great for when time is of the essence. In addition, contrast substances can be injected into the bloodstream to improve the visibility of particular structures or abnormalities, such as tumors or blood arteries, in CT imaging 126. Patients still run the slight risk of radiation-induced side effects from being exposed to ionizing radiation during CT imaging, notwithstanding the benefits 127.

**Figure 7:**
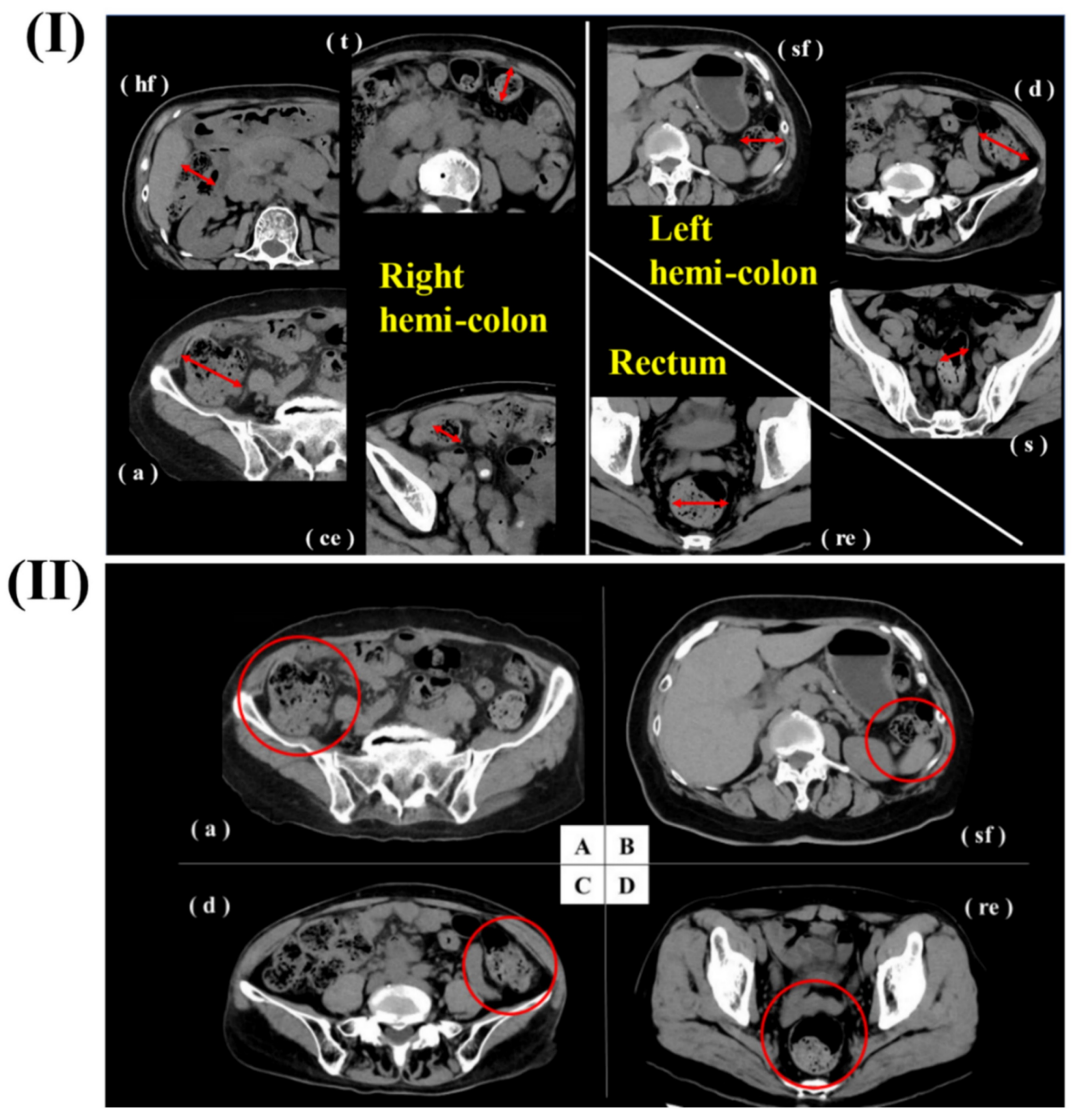
(I) A straightforward CT scan of the abdomen and pelvis has been obtained at each location of the right hemicolon, left hemicolon, and rectum. In the most dilated part of the intestine, which is represented by the red line, the diameter was measured (II) Representative cases. a) The stool volume was rated as four, and the gas volume was rated as two (shown by the red circle) using the CT scan. B) According to the CT picture, the volume of the stool was rated as 1, while the volume of the gas was rated as 2 (red circle). C) The stool volume was rated as three, and the gas volume was rated as two (shown by the red circle) using the CT scan. d) Based on the CT picture, the stool volume was rated as three, and the gas volume was also rated three (the red circle). Reproduced with the permission from ref 128. Fig.1, and Fig.2. (MDPI©2023).

In a study, Haraikawa *et al.* investigated the clinical assessments of PPIs/PCABs for gastric disease. They examined the relationship between the lateral diameter of the colon and the volume of intraluminal contents as estimated from CT images, as well as the symptoms of chronic constipation and stool consistency. Retrospective selection was done on consecutive patients who had simple abdominal CT, the Constipation Scoring System (CSS), and the Bristol Stool Form Scale (BSFS) questionnaires. At each location, the diameter of the intestines was measured, and gas and stool quantities were assessed at five different levels. The average age of the 149 people who took part in the study was 72.1 years old, and there were 54 men and 95 women. There was a significant correlation between gas volume and CSS5 (Time) in the right hemi-colon. Gas volume in the rectum and the BSFS had a negative correlation with each other and with the right hemi-colon and diameter, respectively. Some constipation symptoms and stool consistency were associated with CT findings, which included stool volume, gas volume, and diameter. The evaluation and treatment of constipation may benefit from these results. For every patient, they measured the most dilated sites of each segment of the intestines (c, a, hf, t, sf, d, s, re). They measured the intestinal contents (gas volume and stool volume) for each of the maximal intestinal diameters that were found. One was "none" or "nearly none," two was "less," three was "normal," four was "slightly more," and five was "more" in terms of content rating. As a result of the area of control for blood flow, they classified the segments as either rectum, left hemi-colon (sf + d + s), or right hemi-colon (c + a + hf + t). A right hemi-colon was created by averaging c, a, hf, and t, while a left hemi-colon was created by averaging sf, d, and s (Fig 7) 128.

**Figure 8:**
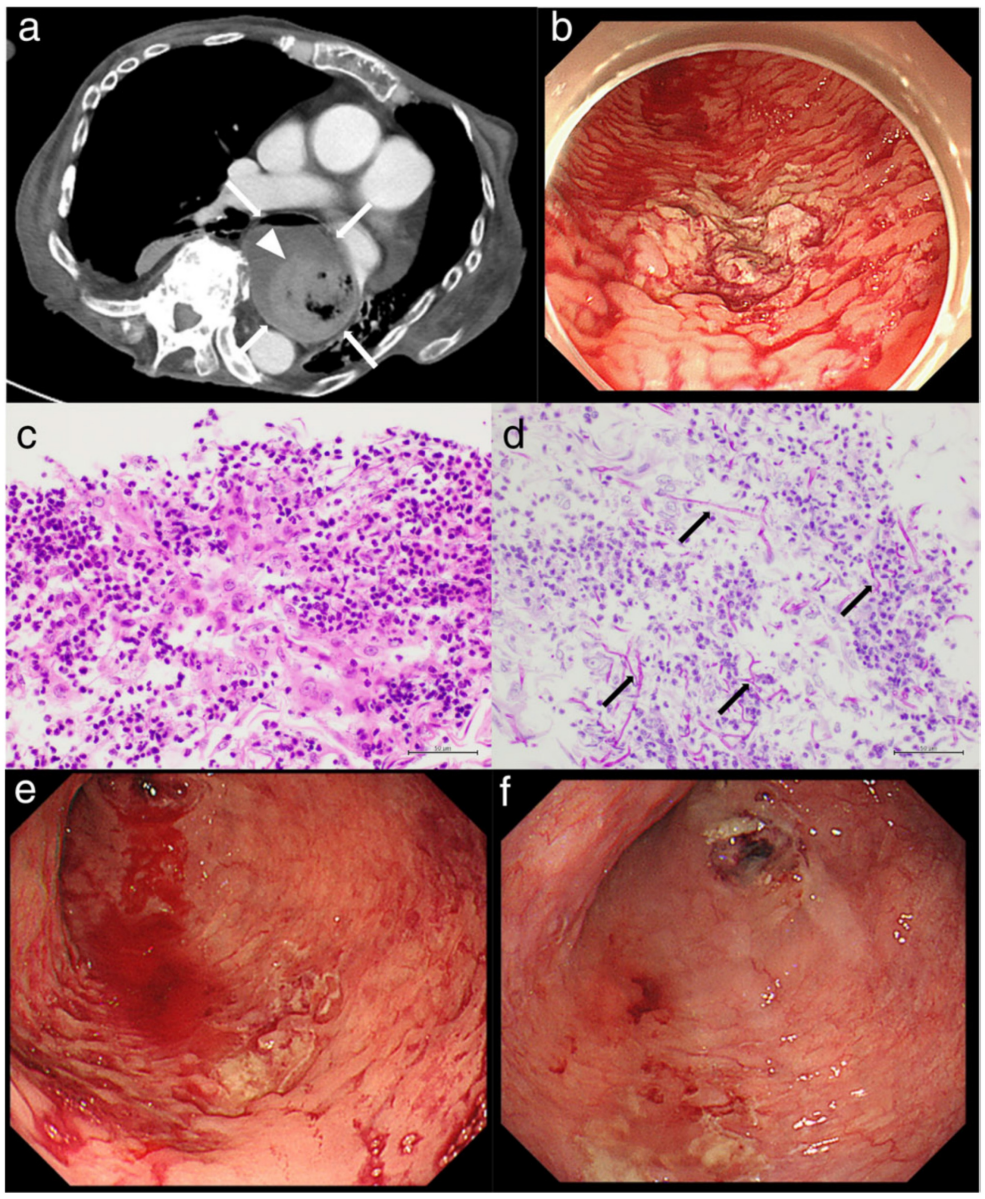
There are three types of imaging: CT, esophagogastroduodenoscopy (EGD), and pathologic pictures. (a) In the CT scan, the esophagus is shown to be highly dilated (white arrows), and it contains both liquid and blood components (white arrowhead). (b) the electrogram reveals several ulcers, both large and small, that are covered in white plaques. (c, d) the esophageal mucosa has been severely destroyed, and lymphocyte invasion has occurred, resulting in the appearance of infiltrated mycelium (black arrows). (e) EGD reveals that the esophageal ulcers have reextravasated and are leaking. (f) After the treatments, the esophageal ulcers are shown to have improved on the EGD. Reproduced with the permission from the ref. 129. Fig.1 (Wiley©2020).

In another study, Natsui *et al.*, investigated the case study of a 64-year-old woman suffering from rheumatoid arthritis (RA) and systemic sclerosis (SSc). She was prescribed prednisolone and abatacept for RA and SSc, and she was also prescribed vonoprazan to prevent steroid-induced gastric ulcers. Pathological examinations and esophagogastroduodenoscopy confirmed a diagnosis of severe Candida esophagitis, characterized by numerous ulcers (both large and small) that were bleeding. The esophageal ulcers showed signs of improvement. Although severe Candida esophagitis is uncommon, it should be remembered that individuals with impaired immune systems and esophageal motility problems, such SSc, are at increased risk of developing this condition. Severe esophagitis can be prevented with regular endoscopic follow-up and preventative medication. Albumin level of 2.3 g/dL (normal range: 4.1-5.1 g/dL) and haemoglobin level of 7.5 mg/dL (normal range: 11.6-14.8 mg/dL) were among the laboratory findings that indicated anaemia and malnutrition in the previous hospital. The results of the CT showed that the esophagus was significantly enlarged and contained both blood and fluids (Fig 8a). During the esophagogastroduodenoscopy procedure, numerous ulcers, both large and tiny, were identified with white plaques. The lesion on the upper posterior wall showed signs of extravasation and oozing. Fig 8b shows that the surrounding mucosa had a cobblestone appearance due to swelling. A biopsy was taken from the ulcer lesions to determine the origin of the esophageal ulcers. The esophageal mucosa was extensively damaged with lymphocyte invasion and infiltrated mycelium was seen in the results of periodic acid-Schiff staining and hematoxylin and eosin staining (Fig 8c,d). These findings led to the conclusion that the esophageal ulcers were caused by Candida esophagitis, which is classified as grade III according to the Kodsi system. After using cauterization to treat the extravasation, they had to do it again since oozing and extravasation kept happening (Fig 8e). The regimen consisted of 200 mg of miconazole and 300 mg of amphotericin B taken orally once a day, followed by 50 mg of micafungin given intravenously. They also started antifungal treatment and whole parenteral nutrition shortly after admission and stopped abatacept. Esophageal ulcers showed improvement with these treatments (Fig 8f) 129.

### Fluorescence imaging for gastric acid related disorders

Fluorescence imaging has revolutionized the diagnosis and management of gastric acid-related disorders by offering enhanced visualization capabilities. This technology allows physicians to detect ulcers, inflammation, or other mucosal abnormalities in the stomach by using fluorescent dyes that bind specifically to certain molecules or tissues. Particularly helpful for detecting minor changes symptomatic of disorders like gastritis or peptic ulcers, endoscopic methods that use fluorescence imaging allows gastroenterologists to do real-time evaluations of stomach mucosa during examinations 130. Furthermore, disorders like GERD can be evaluated with great precision using fluorescent pH-sensitive dyes, which enable the monitoring of gastric acidity levels. Research into gastric physiology, including acid-secreting cell activity and therapeutic intervention effects, is made possible by fluorescence imaging, which goes beyond diagnostics. These methods hold great potential for improving diagnostic accuracy and guiding personalised treatment options for stomach acid-related illnesses. With the help of emerging technologies, they will ultimately lead to better clinical results and patient care in the field of gastroenterology 131.

**Figure 9:**
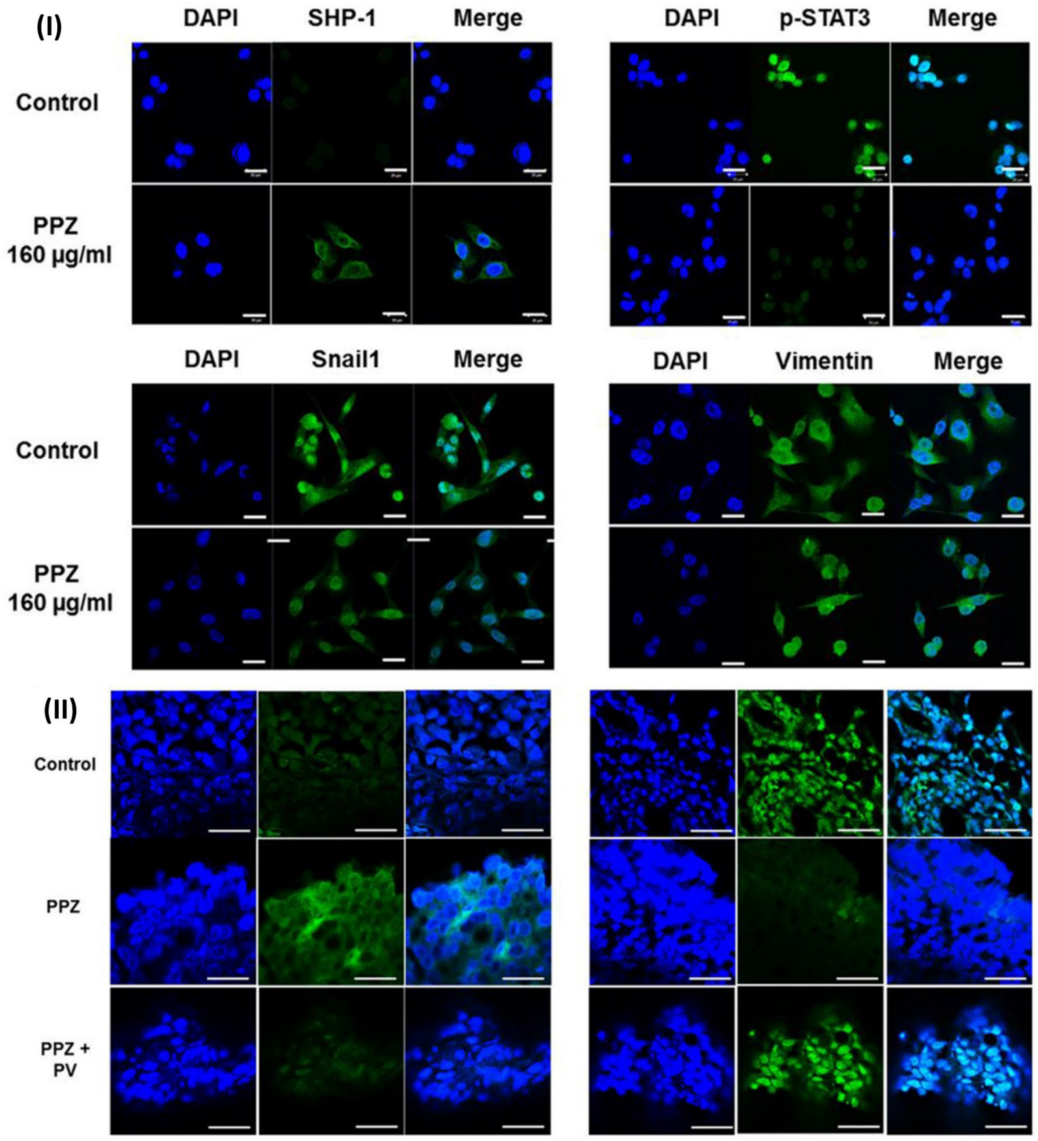
(I) The immunofluorescence of SHP-1 and p-STAT3 following treatment with pantoprazole for 48 hours. (II) Immunofluorescence. Reproduced with the permission form ref 132. Fig. 2 and Fig. 7 (Springer Nature©2018).

In a study, Koh *et al.*, evaluated the effect of pantoprazole for gastric cancer. Because of the decreased expression of SHP-1 and the maintained level of p-STAT3, they utilised AGS and MKN-28 cells. Validation of the role of SHP-1 was accomplished through the utilisation of pharmacologic inhibitors of SHP-1 as well as siRNA. The researchers observed that the expression of SHP-1 was increased by pantoprazole at doses of 40, 80, and 160 μg/ml, while the expression of p-STAT3 was decreased in a dose-dependent way in AGS and MKN-28 cells. Furthermore, pantoprazole reduced migration and invasion of AGS and MKN-28 cells, as well as considerably downregulated mesenchymal markers (Snail1 and vimentin). It also significantly increased epithelial indicators (E-cadherin). They performed pharmacologic inhibition (pervanadate) or knockdown of SHP-1 prior to pantoprazole treatment in order to validate the role of SHP-1 in the inhibition of STAT3 activity by pantoprazole in gastric cancer cells. This resulted in a significant reduction in the suppression of p-STAT3 as well as the anti-migration and invasion effect that pantoprazole had on AGS cells. The intraperitoneal injection of pantoprazole resulted in a considerable reduction in the volume of the tumour in the xenograft tumour model. This was accompanied by an increase in the expression of SHP-1 and a decrease in the expression of p-STAT3, both of which were mitigated by the concurrent injection of pervanadate. Inducing SHP-1 in gastric cancer cells may be the mechanism by which pantoprazole exerts its inhibitory effect on cellular migration and invasion. They carried out immunofluorescence labelling to identify the impact of pantoprazole on the expression of SHP-1, p-STAT3, and EMT markers in AGS cells. Upon treatment with pantoprazole, the results demonstrated an increase in the cytosolic staining of SHP-1, while the nuclear staining of p-STAT3 was reduced in AGS cells. Additionally, the results demonstrated a modification of EMT indicators, namely an increase in the expression of E-cadherin and a decrease in the expression of vimentin and Snail1 (Fig.9I). They noticed that intraperitoneal injection of pantoprazole increased SHP-1 expression and decreased p-STAT3 expression in comparison to the control; however, concurrent administration of pervanadate mitigated this effect. This was seen through the use of immunohistochemical staining. This was further demonstrated by immunofluorescence labelling, which revealed that pantoprazole increased the cytosolic staining of SHP-1 while simultaneously decreasing the nuclear staining of p-STAT3. It was shown that the administration of pervanadate in conjunction with pantoprazole was able to counteract these effects (Fig. 9II). According to the findings of the study, the anti-tumorigenic impact of pantoprazole may be mediated via the modification of the SHP-1/p-STAT3 axis in the context of gastric cancer 132.

In another study, Alai *et al.*, developed lansoprazole nanoparticles for gastric ulcers. This work aims to treat acid reflux problems by combining nanoparticle design with enteric coating technology to sustain the delivery of the acid-labile medication lansoprazole (LPZ). Eudragit® RS100 nanoparticles (ERSNP-LPZ) and poly (lactic-co-glycolic acid) nanoparticles (PLGANP-LPZ), were synthesized via solvent evaporation/extraction. Caco-2 cells were used to evaluate the impact of nanoparticle charge and permeability enhancers on lansoprazole uptake. The confocal microscopy pictures showed that the nanoparticles were successfully delivered to the Caco-2 cell cytoplasm. While sodium caprate improved the transcellular route for negatively charged PLGA nanoparticles, positively charged Eudragit nanoparticles had a far greater rate of cellular uptake. *In vitro* studies showed that both nanoparticle kinds released their drugs continuously. After 7 days of treatment with EC-ERSNP1010-Na caprate or EC-PLGANP1005-Na caprate, respectively, 92.4% and 89.2% of gastric ulcers in ulcer-induced Wistar rats were healed, owing to the sustained and prolonged LPZ concentration achieved by enteric-coated capsules filled with nanoparticles 133.

## Insights into The Reported Case Studies of PPIs and Vonoprazan

The safety and effectiveness of Vonoprazan in real-world settings can be better understood from the reported case studies 134.

To better understand the value of Vonoprazan in varied patient populations and clinical contexts, these studies often supplement findings from controlled clinical trials. The efficacy of vonoprazan in treating refractory instances of GERD, PUD, and Helicobacter pylori infection has been shown in case studies 135. The frequently describe cases where traditional treatments, including PPIs failed to alleviate symptoms or were too painful, and then vonoprazan was able to restore the mucosal tissue and symptoms. Furthermore, case studies provide insight into the possible function of vonoprazan in intricate clinical scenarios, such as the management of GERD in individuals who have obesity and diabetes, or who necessitate acid suppression medication for an extended period 136. In addition, by documenting adverse events observed in real-world practice, case reports help us understand vonoprazan safety profile. These findings are vital for clinical decision-making and the detection of uncommon or unexpected adverse effects. Considerable real-world evidence concerning the effectiveness, safety, and therapeutic value of vonoprazan across a range of gastric acid-related disorders has been provided by documented case studies 137. Together, they supplement results from clinical trials and aid physicians in making educated judgments about each patient's therapy 138.

The facts of a case of severe GERD leading to oesophageal stricture in a 68-year-old lady who started vonoprazan treatment was reported by Miwa *et al.* Numerous lesions with a white globe appearance appeared in every region of her stomach (except the antrum) during the follow-up endoscopy performed one year after she started using vonoprazan. A 70-year-old man who had been treated with vonoprazan for three years had noncancerous stomach lesions discovered at an annual follow-up. Cystic gland dilatations harbouring eosinophilic material were the lesions in both cases. Both patients did not show signs of autoimmune atrophic gastritis. Based on their research, these lesions in the stomach that are not malignant may be linked to vonoprazan 139.

Moreover, Haruma *et al.* compare the safety of vonoprazan and lansoprazole over the long term as a maintenance treatment for healed erosive oesophagitis. After an endoscopy confirmed erosive oesophagitis, patients were randomly assigned to take either 30 mg of lansoprazole once a day or 20 mg of vonoprazan once daily for 4 to 8 weeks while their throats healed. Following endoscopic confirmation of recovery, patients began a 260-week maintenance phase on a once-daily dose of either lansoprazole (15 mg) or vonoprazan (10 mg). The main result was an alteration in stomach mucosal histology. After 208 patients (139 for vonoprazan and 69 for lansoprazole) reached the healing phase, 202 patients (133 for vonoprazan and 67 for lansoprazole) reached the maintenance phase. Among those who were treated with vonoprazan, 109 stayed on treatment for 3 years, while 58 were given lansoprazole. At week 156 of the maintenance phase, histopathological assessment of the stomach mucosa revealed that, compared to lansoprazole, vonoprazan was associated with a higher prevalence of hyperplasia of parietal, foveolar, and G cells. From week 48 to week 156, the vonoprazan group did not have a significantly higher incidence of parietal, foveolar, and G cell hyperplasia. No malignant alterations were seen in either group when the stomach mucosa was evaluated BY histopathological. There were no newly discovered safety concerns. After three years of maintenance treatment with vonoprazan or lansoprazole, no new safety problems were found in Japanese patients with healed erosive oesophagitis, according to an interim VISION analysis 140.

**Figure 10:**
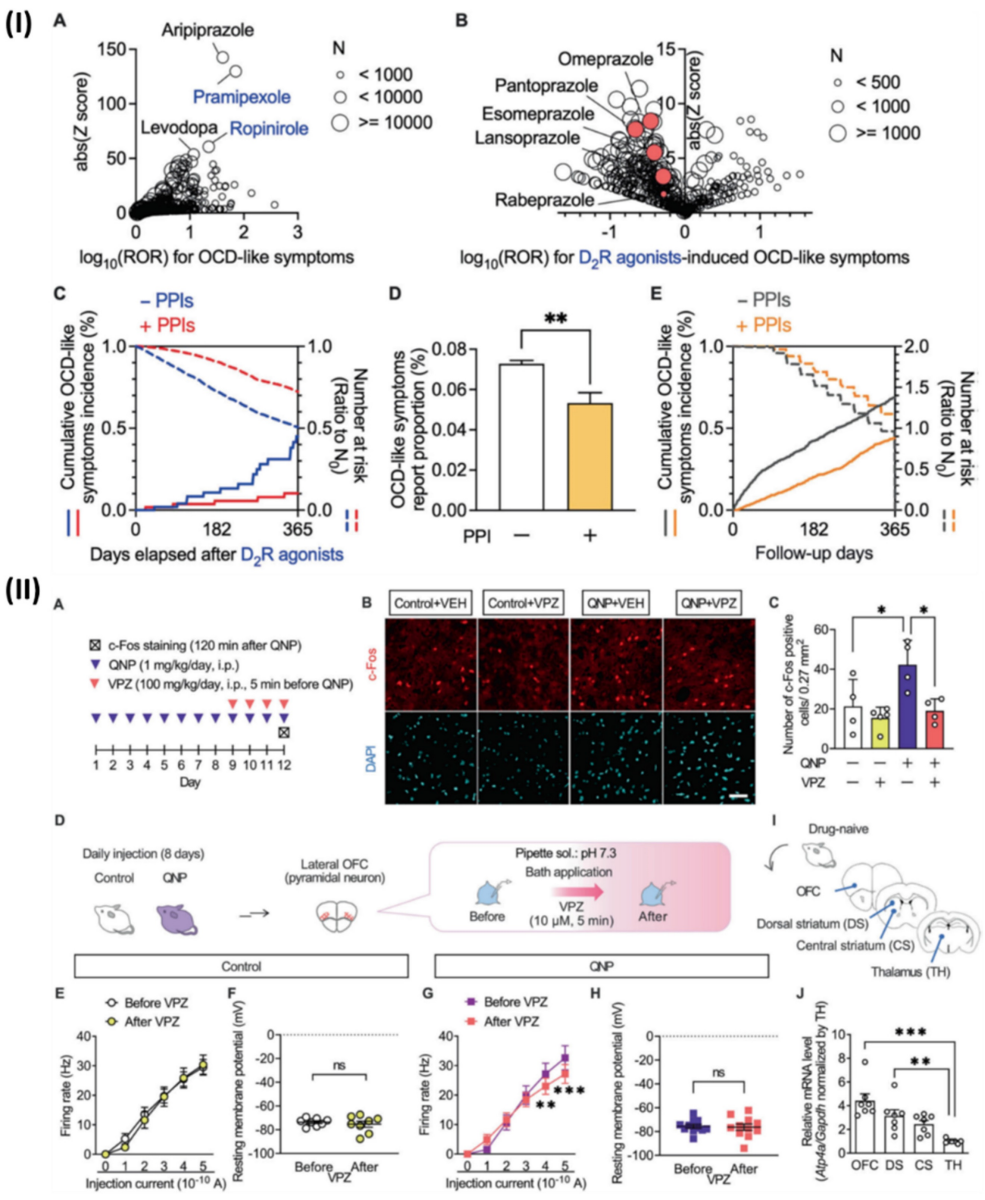
**(I)** The concurrent use of PPIs was found to reduce the occurrence of obsessive-compulsive disorder (OCD)-like symptoms, according to analyses of data from the FAERS and Market Scan. **(II)** The administration of vonoprazan was able to reduce hyperactivity in pyramidal neurons of the lateral OFC that were acquired from mice that had been treated with QNP. Reproduced with the permission form ref 141. Fig.1, and Fig.4 (Springer Nature©2024).

Although, Hatakama *et al.*, analysed a novel therapeutic target for OCD. Using PPIs alongside dopamine D2 receptor (D2R) agonists reduced the occurrence of obsessive-compulsive disorder (OCD)-like symptoms, which were otherwise amplified. In addition, quinpirole, a D2R agonist, caused recurrent and habitual behaviors similar to obsessive-compulsive disorder (OCD) in mice. On the other hand, short-term PPI medication decreased these aberrations. The lateral orbitofrontal cortex is highly expressed by the P-type proton pump gene Atp4a, and in mice that were given quinpirole, PPI reduced the hyperactivity of pyramidal neurons in this area. In primary cultured cortical neurons, Atp4a knockdown mirrored the effects of short-term PPI therapy, which reduced firing activity and intracellular pH. Their research suggests that blocking P-type proton pumps could be an innovative way to treat obsessive-compulsive disorder. Associated with substance abuse and symptoms similar to obsessive-compulsive disorder. Fig 10IA shows that a disproportionality analysis showed that there was a substantial correlation between using dopamine receptor stimulants and more occurrences of symptoms similar to obsessive-compulsive disorder. Dopamine receptor stimulants with high reported odds ratios (ROR) and Z scores included ropinirole and pramipexole, both of which are D2R agonists. Additionally, there was a high correlation between the development of OCD-like symptoms and aripiprazole, a medication that is frequently prescribed as an adjunctive treatment for individuals with treatment-resistant OCD. After carefully examining FAERS data, we found that D2R agonist use was associated with OCD-like symptoms. They looked at Fig 10IB to see how each drug combination affected the results of the confounding analysis for D2R agonists. To determine whether PPIs inhibited D2R agonist-induced OCD symptoms, they conducted a chronological sequence analysis. The hazard ratio for OCD-like symptoms caused by D2R agonists was 0.432. Additionally, they looked at individuals who did not have access to D2R agonists to see if PPI treatment alleviated OCD-like symptoms. Non-D2R agonist users of PPIs had a markedly lower incidence of OCD-like symptoms, according to FAERS data analysis (Fig 10ID). In addition, PPIs were found to significantly reduce the occurrence of OCD-like symptoms in matched cohorts of non-D2R agonist consumers of Market Scan data, with a hazard ratio of 0.533 (Fig 10IE). The effects of PPIs were shown to be due to their action on the brain in the intracerebroventricular injection trials. Hyperactivity in the OFC is a symptom that only SSRI-responsive patients with OCD experience relief from. Hence, they postulated that PPIs influence OFC neuronal activity. Consequently, they used immunostaining to look at how vonoprazan (100 mg/kg) affected the expression of c-Fos, a marker of neuronal activity, in the lateral OFC (Fig 10IIA). The lateral OFC of mice treated with QNP had a higher concentration of c-Fos-positive cells. The opposite was true for animals treated with QNP; injections of vonoprazan reduced the number of c-Fos positive cells (Fig. 10IIB, 10IIC). Additionally, they used *ex vivo* patch clamp recordings to test how vonoprazan affected the activity of neurons in the lateral OFC pyramid (Fig 10IID). Fig 10IIE, 10IIF show that the current-induced firing responses in lateral OFC pyramidal neurons were unaffected by the 10 μM bath vonoprazan administration when compared to control animals. Nevertheless, when mice were treated with vonoprazan, these side effects were considerably reduced (Fig 10IIG, 10IIH). The late period of the depolarizing pulse was when vonoprazan's inhibitory effects on firing response were most noticeable. Since OCD patients often have changed functional connections within the CSTC-loop, they used real-time qRT-PCR to examine the levels of Atp4a mRNA expression in the brain regions associated with the CSTC-loop (Fig 10II(I)). The expression levels of Atp4a mRNA were greater in the OFC compared to the thalamus in drug-naive mice (Fig 10IIJ). Vonoprazan reduced neuronal hyperactivity in the lateral OFC of mice treated with QNPs, as shown here, by blocking P-type proton pumps 141.

## Clinical trials of PPIs and Vonoprazan for Gastric Acid Related Disorders

A newly developed drug that shows promise in the management of gastric acid-related illnesses is vonoprazan. The findings of the clinical trials that tested its effectiveness and safety were quite encouraging. Vonoprazan outperformed more traditional PPIs such as omeprazole and lansoprazole in terms of acid suppression in these trials 142. An appealing alternative for illnesses including peptic ulcer disease, Helicobacter pylori eradication therapy, and gastroesophageal reflux disease is its fast onset of action and persistent acid suppression 143. In addition, vonoprazan helped alleviate symptoms and improve the quality of life for patients with refractory GERD 144. Its ability to effectively reduce stomach acid output regardless of when food is consumed allows for more leeway in dosage schedules. Vonoprazan has a safety profile that is consistent with or lower than that of conventional PPIs, suggesting that it is generally well-tolerated 145. Headache, diarrhoea, and stomach pain are some of the most common side effects, while serious adverse reactions are very unusual. Vonoprazan has shown promise as a treatment for gastric acid related disease in clinical trials due to its effectiveness, fast onset of action, persistent acid suppression, and favourable safety profile. To further understand its safety and effectiveness over the long run, especially when compared to current treatments, more research is required. PPIs have been explored in clinical trials for treating GERD, peptic ulcers, Zollinger-Ellison syndrome, and other illnesses. Studies comparing PPIs to placebo or other acid-reducing therapies show benefits in symptom relief, ulcer healing rates (gastric and esophageal), and avoiding acid-related damage. Additionally, researchers assess pharmacological hazards, such as kidney issues, fractures, and Clostridium difficile infections. Overall, PPIs effectively reduce acid levels, although long-term consequences and optimal use are still being studied. The list of clinical trials of vonoprazan for gastric acid related disease is shown in table 2.

## Conclusion

Proton pump inhibitors and vonoprazan both work to lower gastric acid production, although they do it in distinct ways. Permanently decreasing acid output, PPIs block the gastric parietal cell hydrogen/potassium adenosine triphosphatase enzyme system. Alternatively, vonoprazan is a potassium-competitive acid blocker that, in comparison to PPIs, blocks the last step of acid generation more quickly and effectively. The effectiveness of vonoprazan, a new acid suppressor, in the management of GERD has been studied. When it comes to treating GERD, vonoprazan is on track with or even better than PPIs, according to several clinical trials. The management of disorders connected to gastric acid can be improved through the integration of imaging modalities with clinical results. This allows for a more comprehensive approach, which in turn ensures that patients are diagnosed quickly and treated appropriately. When ulcers or other conditions lead to an imbalance in stomach acid, vonoprazan has shown to be an effective and, in some cases, preferred substitute for traditional PPI treatment. Pharmaceutical formulations of PPIs and vonoprazan incorporate multiple delivery mechanisms to meet the diverse needs of patients. Typical components of such formulations include nanoparticles, a delayed-release formulation, and oral tablets or capsules. This medication's efficacy is ensured by its formulation, which releases it in the intestines instead of the stomach. Additional research is needed to determine its function in cost-efficiency analyses. Essential medicines for the management of problems related to gastric acid include PPIs and vonoprazan, which effectively relieve symptoms, promote mucosal repair, and reduce the risk of consequences. PPIs and vonoprazan, used to treat acid-related gastrointestinal disorders, are being investigated for application in sophisticated micro/nano drug delivery systems. These PPIs reduce stomach acid and improve sensitive drug stability and bioavailability. They prevent medication degradation in the stomach by making it less acidic, enabling more efficient transport to target areas. Their capacity to control medication release allows precise targeting in the gastrointestinal tract, boosting therapy efficacy and reducing side effects. Coupled nanoparticles of PPIs and vonoprazan allow drug release under specific physiological situations, such as tumours' slightly acidic environment. They also have potential in imaging applications for real-time medication distribution and release. By lowering therapeutic doses and adverse effects, PPIs and vonoprazan in drug delivery systems increase therapy efficacy and safety. Physicians prescribe PPIs and vonoprazan based on specific gastrointestinal conditions. PPIs are commonly recommended for treating conditions like GERD, peptic ulcer disease, H. pylori infections, erosive esophagitis, Zollinger-Ellison syndrome, and for the prophylaxis of NSAID-induced ulcers. They work by reducing stomach acid, with treatment durations typically ranging from 4 to 8 weeks, though chronic conditions may require longer use.

Vonoprazan is often prescribed in cases where rapid and sustained acid suppression is needed or when patients do not respond well to PPIs. It is used for similar conditions, including GERD, H. pylori eradication, peptic ulcer disease, and erosive esophagitis. The choice between PPIs and vonoprazan depends on factors such as the severity of the condition, patient response to treatment, side effect profiles, and cost considerations. Regular monitoring is essential to assess treatment efficacy and manage any potential side effects, especially in long-term use. Healthcare practitioners have more flexibility to customise treatment to each patient's needs due to the variety of their formulations and modes of action. To minimise the hazards of long-term use and achieve the best possible treatment results in managing these common gastrointestinal disorders. When considering the use of PPIs and vonoprazan, it is important to use clinical expertise. Patients with acid-related gastrointestinal illnesses, including peptic ulcer disease and GERD, may benefit from taking vonoprazan in conjunction with PPIs. Because of its unique action mechanism and the quicker start of acid suppression, vonoprazan may be useful when PPIs alone are ineffective. Nevertheless, there is an absence of clinical research that has looked at the synergistic effects of PPI and vonoprazan combinations. Healthcare practitioners may weigh the benefits of this combination against the risks and side effects of long-term acid suppression medication, taking into account each patient's reaction and the severity of their ailment.

## Figures and Tables

**Table 1:** Pharmaceutical formulations containing Vonoprazan and PPIs for gastric acid-related disease

Formulations	Drug name	Dose	Clinical outcomes	Ref.
Delayed-Release Tablets	Dexlansoprazole	30 mg	A single dexlansoprazole capsule containing 60 milligrams was equivalent to the 24-hour intragastric pH regulation that was achieved after the administration of two dexlansoprazole 30 mg oral dosage tablets. ODT and pill were both well tolerated by the patients.	54
Delayed-Release Tablets	Esomeprazole magnesium	160 mg	A single unit dosage of the prepared formulation can be utilized for the treatment of acid-related disorders. This formulation can circumvent the process of esomeprazole degradation that occurs through the enteric coating technique. As a result, a formulation of Esomeprazole delayed-release tablet that is both pharmaceutically similar and robust was produced.	55
Delayed-Release Capsules	Omeprazole	20 mg	Reported differences in *in vitro* performance between the brand-name and generic omeprazole delayed-release capsules have not been borne out by the available scientific data.	56
Minitablets	Esomeprazole Magnesium	40 mg	The dual-release properties of dual-release polycap make it a viable alternative to commercial goods that raise the bar for NAB and dosage compliance.	57
Minitablets	Esomeprazole	40 mg	The minitablets and Nexium were found to be bioequivalent in *in vivo* experiments conducted under fasting conditions involving 22 people. Food significantly delays the absorption of medication from minitablets, according to a fed trial that included 16 patients.	58
Pellets	Rabeprazole sodium	20 mg	The current study aimed to protect albino Wistar rats from stomach ulcers caused by 70% ethanol by designing, formulating, and characterizing Rabeprazole enteric coated pellets utilizing the dry powder layering method.	59
Pellets	Omeprazole	20 mg	The purpose of this research was to find out how commercial omeprazole pellets fared when exposed to a pH level above neutral.	60
Bilayer Tablets	Lansoprazole, Amoxicillin	15, 500 mg	Improved patient compliance towards appropriate ulcer care was achieved with the use of bilayer tablets containing lansoprazole and amoxycillin.	61
Bilayer Tablets	Clarithromycin, Esomeprazole	250, 20 mg	Clinical practice can benefit from the use of controlled-release effervescent floating bilayer tablets by reducing dosing frequency, increasing patient compliance with continuous drug release for 24 hours, and possibly improving therapeutic efficacy.	62
Mucoadhesive tablets	Omeprazole	20 mg	In order to reduce dosage frequency and dose-related side effects, increase bioavailability, and avoid the first-pass effect, this study formulated and evaluated mucoadhesive buccal tablets containing the PPI medicine omeprazole.	63
Mucoadhesive buccal tablet	Pantoprazole sodium sesquihydrate	20 mg	The results show that pantoprazole buccal tablet stability in saliva for at least 6 hours and improved oral bioavailability are both caused by magnesium oxide.	64
Mucoadhesive tablets	Omeprazole	20 mg	The current research found that omeprazole pellets with carbopol-934P might be administered orally for controlled release and locally for ulcer disease.	65
Tablets	Vonoprazan	20 mg	For individuals with non-erosive reflux disease, on-demand medication with 20 mg vonoprazan was just as effective as continuous PPI maintenance therapy.	66
Tablets	Lansoprazole, Vonoprazan	30, 20 mg	UBT was found to be less effective when administered with VPZ, suggesting that patients undergoing VPZ medication should undergo thorough evaluation of UBT.	67
Enteric coated tablets	Pantoprazole	40 mg	A model of stress-induced ulcers caused by water immersion was used to assess the anti-ulcer activity. There was a marked decrease in ulcer development with the enteric coated pantoprazole pills.	68

**Table 2:** Clinical trials of Vonoprazan and PPIs for Gastric Acid Related Diseases

Title of the study	Phases	Study type	Status	NCT number
The VATH-1 Study on the Effectiveness of Daily 20-Mg Vonoprazan in Treating Helicobacter Pylori Infection	Phase 4	Interventional	Recruiting	NCT05590286
Evacuation of Helicobacter pylori by Dual and Triple Therapies Based on Vonoprazan	Phase 3	Interventional	Completed	NCT02827942
An RCT on Vonoprazan for the Treatment of Helicobacter Pylori	Phase 4	Interventional	Completed	NCT04901663
Research on the Concentrations of Vonoprazan in Breast Milk from Healthy Breast-Feeding Mothers Who Receive 20 mg of Vonoprazan Daily	Phase 1	Interventional	Recruiting	NCT06391177
Triple Therapy with Bismuth, Amoxicillin, and Vonoprazan, Dual Therapy with Vonoprazan and Amoxicillin, and Standard Triple Therapy Based on PPIs for the Eradication of HP	Not Applicable	Interventional	Recruiting	NCT06349486
Treatment with Vonoprazan Competing with the Gold Standard in Helicobacter Pylori Treatment	Phase 4	Interventional	Recruiting	NCT05614934
The Effectiveness of Vonoprazan in Preventing Ulcers Following Variceal Band Ligation	Phase 3	Interventional	Completed	NCT05227833
Improving Helicobacter Pylori Treatment with a Dual Approach Using Vonoprazan	Phase 4	Interventional	Recruiting	NCT05649540
Improving the Efficiency of Vonoprazan and Amoxicillin for the Elimination of Helicobacter Pylori Infection	Phase 3	Interventional	Completed	NCT05719831
Monitoring the Use of Vonoprazan for the Treatment of "Gastric Ulcer, Duodenal Ulcer, and Reflux Esophagitis"	Not Applicable	Observational	Completed	NCT03214952
Improving the Efficiency of Vonoprazan and Amoxicillin for the Elimination of Helicobacter Pylori Infection	Phase 3	Interventional	Completed	NCT05719831
A Clinical Investigation of Vonoprazan in China	Not Applicable	Observational	Completed	NCT04501627
Evacuation of Helicobacter pylori by Dual and Triple Therapies Based on Vonoprazan	Phase 3	Interventional	Completed	NCT02827942
The effects of two different forms of omeprazole (P07812) were compared in a pharmacodynamic study	Phase 3	Interventional	Completed	NCT01077076
There was a comparison of the pharmacodynamic effects of Zegerid® and Prilosec OTC® in the study CL2008-02 (P07814)	Phase 3	Interventional	Completed	NCT00765206
Omeprazole or Pantoprazole given intravenously to patients with gastric acid hypersecretion	Phase 2	Interventional	Completed	NCT00001191
Randomised Controlled Trial of Pantoprazole for the Treatment of Chronic Acid Peptic Constipation	Phase 3	Interventional	Completed	NCT00261300
